# Equilibrative nucleoside transporter 1 inhibition rescues energy dysfunction and pathology in a model of tauopathy

**DOI:** 10.1186/s40478-021-01213-7

**Published:** 2021-06-22

**Authors:** Ching-Pang Chang, Ya-Gin Chang, Pei-Yun Chuang, Thi Ngoc Anh Nguyen, Kuo-Chen Wu, Fang-Yi Chou, Sin-Jhong Cheng, Hui-Mei Chen, Lee-Way Jin, Kevin Carvalho, Vincent Huin, Luc Buée, Yung-Feng Liao, Chun-Jung Lin, David Blum, Yijuang Chern

**Affiliations:** 1 Institute of Biomedical Sciences, Academia Sinica, Nankang, Taipei, 115 Taiwan; 2 School of Pharmacy, National Taiwan University, Taipei, Taiwan; 3 Neuroscience Program of Academia Sinica, Academia Sinica, Taipei, Taiwan; 4 Department of Pathology and Laboratory Medicine, University of California Davis, Sacramento, CA USA; 5 Univ. Lille, Inserm, CHU Lille, U1172 - LilNCog - Lille Neuroscience & Cognition, 59000 Lille, France; 6 Alzheimer & Tauopathies, LabEx DISTALZ, LiCEND, 59000 Lille, France; 7 Institute of Cellular and Organismic Biology, Academia Sinica, Taipei, Taiwan

**Keywords:** Alzheimer’s disease, Tauopathy, Adenosine, AMPK, ENT1

## Abstract

Tau pathology is instrumental in the gradual loss of neuronal functions and cognitive decline in tauopathies, including Alzheimer’s disease (AD). Earlier reports showed that adenosine metabolism is abnormal in the brain of AD patients while consequences remained ill-defined. Herein, we aimed at investigating whether manipulation of adenosine tone would impact Tau pathology, associated molecular alterations and subsequent neurodegeneration. We demonstrated that treatment with an inhibitor (J4) of equilibrative nucleoside transporter 1 (ENT1) exerted beneficial effects in a mouse model of Tauopathy. Treatment with J4 not only reduced Tau hyperphosphorylation but also rescued memory deficits, mitochondrial dysfunction, synaptic loss, and abnormal expression of immune-related gene signatures. These beneficial effects were particularly ascribed to the ability of J4 to suppress the overactivation of AMPK (an energy reduction sensor), suggesting that normalization of energy dysfunction mitigates neuronal dysfunctions in Tauopathy. Collectively, these data highlight that targeting adenosine metabolism is a novel strategy for tauopathies.

**Supplementary Information:**

The online version contains supplementary material available at 10.1186/s40478-021-01213-7.

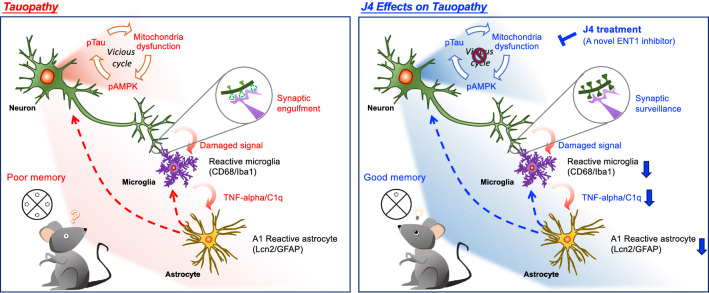

## Background

Alzheimer’s disease (AD) is the most prominent neurodegenerative disease in aging societies, but there is no effective treatment [1]. The major pathogenic hallmarks of Alzheimer’s disease include extracellular amyloid plaques (amyloid-beta, Aβ) and intracellular accumulation of neurofibrillary tangles made of hyperphosphorylated Tau. The latter is particularly known to be associated with neuritic dystrophy, synapse loss, and neuroinflammation, which lead to cognitive impairments [2–5]. Phosphorylation of Tau can be modulated by more than 30 kinases and protein phosphatases [6, 7], thus making it very sensitive to the environment including metabolic changes [8, 9].

Adenosine is an important homeostatic building block of many important metabolic pathways. It also serves as a neuromodulator that controls multiple functions (including neuroinflammation, blood–brain barrier permeability, neuronal transmission, and energy balance) through receptor-dependent and/or independent mechanisms in the central nervous system [10, 11]. The main sources of adenosine include ATP catabolism and the transmethylation pathway. In addition, the extracellular and intracellular adenosine levels are modulated through equilibrative nucleoside transporters (ENTs) and concentrative nucleoside transporters [12, 13]. ENTs are bidirectional transporter that transports adenosine in a concentration-dependent, Na^+^-independent manner. These ENT members contain 11 transmembrane domains, and can be found in most cell types (including neurons and astrocytes) in the brain. Among the four ENTs, ENT1 has attracted much attention in recent years because it has the highest affinity for adenosine [14, 15]. Since disruption of adenosine homeostasis in the brain has been implicated in several neurological disorders (including sleep disorders, impaired cognition impairment and neurodegenerative diseases [16, 17]), modulation of adenosine levels by controlling the components of adenosine metabolism therefore is a potential therapeutic approach.

Impaired adenosine homeostasis has been also suggested in the brain of AD patients [18]. In this study, Alonso-Andres et al. had reported that the adenosine levels in the frontal cortices of AD patients were lower than those of age-matched controls throughout disease progression. Conversely, the adenosine levels of parietal cortices gradually become higher than normal subjects at the later stage of AD [18]. No data of the hippocampal adenosine level was reported in this study. These findings overall suggest that adenosine homeostasis may be dysregulated in AD in a brain area-specific manner. Accordingly, dysregulation of the two main adenosine receptors, A_1_ and A_2A_ have been reported in the brain of patients [19, 20] as well as in AD mouse models [21–24].

The link between adenosine homeostasis and Tau pathology remains ill-defined. Interestingly, previous studies have associated AMPK activation with abnormal Tau phosphorylation in the brains of patients and mice with Alzheimer’s disease [25–28]. AMP-activated protein kinase (AMPK) is a homeostatic energy sensor that controls the balance between anabolic and catabolic processes in cells [29]. In the presence of stress (e.g., an elevated AMP/ATP ratio, high reactive oxygen species levels, or mitochondrial dysfunction), AMPK can be activated by being phosphorylated at Thr^172^ on the α subunit of AMPK [30]. Under adverse conditions, for instance when the extracellular adenosine concentration is elevated, transport of adenosine into cells enhances the cellular level of AMP, alters the AMP/ATP ratio, and subsequently activates AMPK [31, 32]. Together, these observations raised the possibility of a link between adenosine homeostasis, AMPK and Tau.

We have addressed this link using the in-house synthetic compound J4. J4 is an orally active, BBB-permeable inhibitor of ENT1 with a Ki value of 0.05 μM [22]. Intrahippocampal acute infusion of J4 elevated the extracellular adenosine level determined by microdialysis in THY-Tau22 and WT mice (Additional file 1: Fig. S1). Its oral bioavailability is 48% in mice with brain-to-blood ratio of approximately 16.4% (Additional file 1: Table S1), indicating that J4 can enter the brain via oral administration. In the present study, we investigated the impact of adenosine homeostasis modulation by J4 in a Tauopathy context, using the THY-Tau22 (Tau22) model, which progressively develops hippocampal Tau pathology and memory deficits [33]. We demonstrated that chronic treatment with J4 mitigates the development of hippocampal Tau pathologies (including synapse loss, mitochondrial dysfunction, and neuroinflammation). These beneficial effects of J4 are particularly ascribed to its ability to suppress AMPK overactivation in the hippocampi of Tau22 mice, suggesting that it normalizes energy dysfunction caused by pathogenic Tau.

## Methods

### Animals and drug administration

THY-Tau22 (Tau22) mice ((B6.Cg-Tg(Thy1-MAPT)22Schd) expressing mutant human 4R Tau (G272V and P301S) driven by a neuron-specific promoter (Thy1.2) were maintained on the C57BL/6J background [33]. All animal studies were conducted following the protocols approved by the Institutional Animal Care and Utilization Committee (IACUC, Academia Sinica, Taiwan). All mice were housed in ventilated cages (IVC) with freely accessible water and chow diet (LabDiet®, San Antonio, TX, USA), and kept under controlled lighting condition with 12:12 h light/dark cycle at the Institute of Biomedical Sciences Animal Care Facility (Taipei, Taiwan). To investigate the effect of J4, male Tau22 mice and their littermate controls were randomly allocated to experimental groups that treated with the J4 (0.02 mg/ml in 1% HPβCD; designated TauJ and WTJ mice) or vehicle (1% HPβCD; designated TauC and WTC mice) continuously in drinking water for 7 months from the age of 3 to 10 months. Males were chosen for the following experiments. The behavioral tests, electrophysiological study, and RNA-seq analysis were carried out at the age of 10 months. The immunohistochemical staining and quantitative PCR were performed at age of 11 months. The phospho-proteomic analysis was assessed at age of 12 months. Mice had continuously received the indicated treatment during experiments.

### Human cases

For immunofluorescence analysis, a total of 18 post-mortem Human posterior hippocampal specimens: six normal subjects, six Alzheimer’s disease, and six FTD-Tau (CBD, PSP, and Pick’s), were obtained from the UC Davis Alzheimer’s Disease Center (USA).

For mRNA analysis, a total of 55 post-mortem Human brain samples (Brodmann area 10 prefrontal cortex or temporal cortex): 13 normal subjects, 19 Alzheimer’s Disease, and 23 FTD-Tau (CBD, P301L, PSP, and Pick’s), were obtained from the brain banks of Lille, Paris, and Geneva. Participants and methods have been described previously [34, 35]. Fresh frozen grey matter tissue (about 100 mg) retrieved at autopsy and stored at − 80 °C was used for mRNA analysis. All the brain samples used for RT-qPCR analyses had an RNA integrity number ≥ 5. Detailed information on the normal subjects, Alzheimer’s Disease patients, and FTD-Tau patients from which the specimens used in the present study were obtained is listed in Additional file 1: Table S2.

### Morris water maze

Spatial memory and cognitive flexibility of mice aged 10–11 months were evaluated using the Morris water maze test as described with slight modifications [36]. A circular swimming pool (154 cm in diameter, 51 cm in height) was filled with milky water (30 cm in depth, kept at 20 °C), and divided into four quadrants (T: target; L: target left; R: target right; O: opposite) with distinct visual cues on the tank wall of each quadrant. The hidden platform (13 cm in diameter, 0.5 cm below the surface of the milky water) was placed in the center of the target quadrant (T). Each mouse underwent four daily training trial (120 s/trial, 30 min interval), in which they were released from randomly selected nontarget quadrants (NTs). For the spatial memory test in the acquisition-learning phase, learning trials were performed with a hidden platform for five consecutive days (Day 1–Day 5). For the spatial reversal memory test in the reversal-learning phase, learning trials were performed with a hidden platform relocated to the opposite quadrant for an additional four consecutive days (Day 9–Day 12). To evaluate reference memory, the probe trial and reversal probe trial were performed on Day 8 (72 h after the acquisition-learning phase) and Day 15 (72 h after the reversal-learning phase), respectively. For the probe test, the hidden platform was first removed. The mouse was then released in the opposite quadrant (O), and their swimming path was recorded for 120 s. The swimming path and other parameters (e.g. escape latency and swimming speed) of each mouse in different quadrants were monitored and analyzed using the TrackMot video tracking system (Diagnostic & Research Instruments Co., Ltd., Taoyuan, Taiwan). Mice that exhibited nonsearching behaviors (floating, a swimming speed below 10 cm/s and circling) were excluded from the analysis. Statistical differences were analyzed by two-way ANOVA.

### Electrophysiological study

Mice aged 10–11 months were used for electrophysiology approaches. All electrophysiology studies were performed at the electrophysiology core facility (Neuroscience Program of Academia Sinica, Taipei, Taiwan). After rapid decapitation, the hippocampus was quickly dissected out and immersed in ice-cold artificial cerebrospinal fluid (ACSF; 119 mM NaCl, 2.5 mM KCl, 2.5 mM CaCl_2_, 1.3 mM MgSO_4_, 1 mM NaH_2_PO_4_, 26.2 mM NaHCO_3_, and 11 mM glucose) oxygenated with 95% O_2_ and 5% CO_2_. Transverse hippocampal slices of 450 μm thickness were prepared with a DSK Microslicer (DTK-1000, Osaka, Japan) filled with oxygenated ice-cold ACSF. For recovery, the slices were then incubated in an interface-type holding chamber filled with oxygenated ACSF at RT for at least 3 h. Before recording, the recording electrodes were prepared with a glass micropipette puller (PC-10, Narishige, Tokyo, Japan), and the slices were transferred to an immersion-type recording chamber equipped with a perfusion system (flow rate: 2–3 ml/min) and temperature controller (kept at 32 °C). To record field excitatory postsynaptic potentials (fEPSPs) recording, the bipolar stainless-steel stimulating electrodes (Frederick Haer Company, Bowdoinham, ME; 10 ΩM impedance) and a glass pipette filled with 3 M NaCl were placed in the stratum radiatum of the hippocampal CA1 region. Basal synaptic transmission at the Schaffer collateral-CA1 synapses was first evaluated by measuring input–output curves using 12 stimuli (constant current pulses from 10 to 120 μA in increments of 10 μA, duration of 40 μs). To measure the paired-pulse facilitation (PPF) response, two pulses were applied in rapid succession (interpulse intervals of 50, 100, 150, 200, 300, 400 and 500 ms). Baseline responses were recorded by applying single stimuli (40 μs pulse-width) at 30 s intervals, and 4 responses were averaged to obtain a data point. Long-term depression (LTD) was induced using 3 trains of low-frequency stimulation (LFS, 1200 pulses at 2 Hz) with a 10-min interval/train as described elsewhere [37]. The initial fEPSP slope was calculated by using Signal software (V4.08, Cambridge Electronic Design, Cambridge, UK).

### Phospho-proteomic analysis

Hippocampal lysates (200 μg) collected from three animals from each condition were subjected to phospho-proteomic analysis at the Proteomics Core Facility (PCF) (Institute of Biomedical Sciences, Academia Sinica, Taipei, Taiwan). For in-solution digestion, 8 M urea was added to the lysates to prepare a mixture of 10 mg protein/ml in 6 M urea. Protein reduction was performed by the addition of dithiothreitol (DTT, final concentration 5 mM) and incubation at 56 °C for 25 min. Protein alkylation was performed by the addition of iodoacetamide (IAA, final concentration 15 mM) and incubation at RT for 45 min to block the reversion of sulfhydryl (–SH) groups to disulfide bonds. Trypsin/Lys-C Mix (Promega, WI, USA) was then added to protein (protein: protease = 25:1 w/w) and the mixture was incubated for 4 h at 37 °C. The urea concentration was adjusted to 1 M or less by diluting the reaction mixture with triethylammonium bicarbonate (TEAB, 50 mM) followed by an incubation at 37 °C for 17 h. The digested samples were dried with a SpeedVac and desalted with C18 Oasis® PRiME HLB cartridges (Waters, MA, USA). For iTRAQ labeling, the digested peptides were labeled with four isobaric iTRAQ Reagents (114, 115, 116, and 117) using iTRAQ® Reagents-4plex Applications Kit (AB Sciex, MA, USA) following manufacturer's instructions. For phosphopeptide enrichment, iTRAQ labeled peptides were mixed with loading buffer (80% acetonitrile, ACN, 5% trifluoroacetic acid, TFA, and 1 M glycolic acid) and adjusted to pH 2. The sample solution was then mixed with TiO_2_ beads (GL Sciences, Japan) and incubated with vortexing at RT for 15 min. The beads were collected and washed twice with 100 μl washing buffer (80% ACN and 5% TFA). The phosphopeptides were sequentially eluted with 50 μl 0.5% NH_4_OH, 50 μl 5% NH_4_OH, and 50 μl 80% ACN with 0.1% formic acid, and dried with a SpeedVac. The phosphopeptides were then subjected to LC/MS/MS and analyzed by Proteome Discoverer ver.2.2 (Thermo Fisher Scientific, Waltham, MA, USA).

### RNA extraction, cDNA synthesis, and quantitative PCR

For mouse brain tissue, RNA isolation and complementary DNA (cDNA) synthesis were performed according to the manufacturer’s protocols. In brief, mouse hippocampal tissues were homogenized in GENEzol™ reagent (GZX100, Geneaid Biotech Ltd., New Taipei City, Taiwan) with sterilized tissue grinders (FocusBio, Taiwan), and then standard procedures for RNA preparation and cDNA synthesis were performed as described previously [22]. Quantitative PCR (qPCR) assays were carried out using the LightCycler® 480 System (Roche Life Science, Indiana, USA) and analyzed by the comparative CT (ΔΔCt) method with GAPDH as a reference gene. The sequences of the PCR primers are shown in Additional file 1: Table S3.

For Human brain tissue, total mRNA was extracted and purified using the RNeasyLipid Tissue Mini Kit (Qiagen). One microgram of total mRNA was reverse-transcribed using the HighCapacity cDNA reverse transcription kit (Applied Biosystems). Quantitative real-time polymerase chain reaction (qPCR) analysis was performed on an Applied Biosystems™ StepOnePlus™ Real-Time PCR Systems using TaqMan™ Gene Expression Master Mix (Applied Biosystems™). The thermal cycler conditions were as follows: 95 °C for 10 min, then 40 cycles at 95 °C for 15 s and 60 °C for 1 min. Amplifications were carried out in duplicate and the relative expression of target genes was determined by the ΔΔCt method with β-Actin (ACTB) was used as a reference housekeeping gene for normalization. References of the probes used in this study are given in Additional file 1: Table S4.

### RNA sequencing (RNA-seq)

Total RNA samples (3 μg per sample) extracted from the hippocampus with RIN values greater than 8 were subjected to RNA-seq analysis. The RNA library construction and sequencing were carried out by Welgene Biotech (Taipei, Taiwan). Briefly, the SureSelect Strand-Specific RNA Library Preparation Kit (Agilent Technology, CA, USA) was used for library construction on the Illumina platform. After AMPure XP Bead-based (Beckman Coulter Genomics, MA, USA) size selection of the RNA library, the sequences were determined using the Illumina’s sequencing-by-synthesis (SBS) technology to obtain 150-bp paired-end reads. Sequencing data (FASTQ files) were generated by Welgene’s pipeline (Base call conversion, adaptor clipping, and sequence quality trimming) based on Illumina’s base-calling program bcl2fastq v2.2.0 (Illumina, CA, USA) and Trimmomatic v0.36 [38]. The RNA-seq reads were then aligned to the mouse reference genome (mm10) from the Ensembl database (Ensembl release 93) using HISAT2 [39]. The sample-to-sample distances were visualized by principal components analysis (PCA) (Additional file 1: Fig. S2). Expression levels (fragments per kilobase per million, FPKM) were analyzed and estimated using cuffdiff (cufflinks v2.2.1) [40] and Welgene in-house programs. To identify the differentially expressed (DE) genes in different groups, cutoff criteria (absolute log_2_ fold change ≥ 0.32, *p* < 0.05) were used. A volcano plot and heatmap of the DE genes were drawn by using Instant Clue [41] and Morpheus software (https://software.broadinstitute.org/morpheus/), respectively. The Gene Ontology (GO) and the Kyoto Encyclopedia of Genes and Genomes (KEGG) pathways of DE genes were analyzed by using the Database for Annotation, Visualization and Integrated Discovery (DAVID 6.7) [42, 43]. The Ingenuity Pathway Analysis (IPA) software (Qiagen, CA, USA) was then used for the identification of canonical pathway(s) related to the DE genes. Z-score was used to predict activation (Z-score ≥ 2.0) or inhibition (Z-score ≤  − 2.0) of the indicated pathway.

### Immunohistochemical staining

Coronal brain sections (20 μm) from the desired mice were prepared as previously described [22]. For immunofluorescence (IF) staining, brain slices were washed with 0.1 M PBS buffer, permeabilized with 0.2% Triton X-100 solution (in 0.1 M PBS buffer), and blocked with 3% normal goat serum (NGS), 3% normal donkey serum (NDS), or 3% bovine serum albumin (BSA) in 0.1 M PBS buffer for 2 h at RT. The brain sections were then washed with 0.1 M PBS buffer twice and incubated with the indicated primary antibodies (listed in Additional file 1: Table S5) in primary antibody solution (1% NGS or BSA, 0.2% Triton X-100, and 0.1% sodium azide in 0.1 M PBS) for 48 h at 4 °C. After extensive washes, the brain sections were incubated with the corresponding secondary antibody (1:500) for 2 h at RT, and then the nuclei were stained with Hoechst 33258 (1:5000) for 10 min at RT. Free-floating brain sections were mounted on the silane-coated slides (Muto Pure Chemicals Co., Tokyo, Japan) with mounting media (Vector Laboratories, CA, USA) and stored at 4 °C before imaging. An LSM 780 confocal microscope (Carl Zeiss, Germany) was used to capture images. The images were analyzed with MetaMorph software (Universal Imaging, PA, USA).

Formalin-fixed, paraffin-embedded human brain slices were deparaffinized and rehydrated. To expose the antigenic sites, the brain slices were then immersed in 1X citrate buffer (C9999, Sigma-Aldrich, St. Louis, MO, USA) for 20 min at 97.5 °C and cooled to RT. The brain slices were subjected to immunofluorescence staining as described above. After secondary antibody (1:500) incubation, the brain sections were treated with 0.1% (w/v) Sudan Black B (199664, Sigma) in 70% ethanol for 15 min at RT to block autofluorescence signal. The brain sections were then stained with Hoechst 33258 (1:5000) for 10 min at RT. After extensive washes with PBS, the brain sections were mounted with the mounting media (Vector Laboratories, CA, USA) and stored at 4 °C before imaging.

### Statistics

The experimental condition was blinded to investigators during behavioral and electrophysiological experiments. The data are expressed as the mean ± S.E.M. All statistical analyses were performed using GraphPad Prism Software (La Jolla, CA, USA). Two-tailed unpaired Student’s *t*-test was used to compare the difference between the two groups. One-way or two-way ANOVA followed by Tukey’s multiple comparisons test was used for comparison of multiple groups. Differences were considered statistically significant when *p* < 0.05.

## Results

### Chronic J4 treatment stabilizes the abnormally altered expressions of genes that control adenosine metabolism

WT and Tau22 male mice were treated with J4 (0.02 mg/ml in 1% HPβCD) or vehicle (1% HPβCD) in drinking water for 7 months from the age of 3 months. The average daily intake level of J4 was 3.06 ± 0.28 mg/kg. The hippocampi of the treated animals were then collected to harvest total RNA for RT-qPCR analysis. The levels of at least three genes (i.e., adenosine deaminase (ADA), CD39, CD73) involved in adenosine metabolism were found elevated in the hippocampus of Tau22 mice, supporting a change in adenosine homeostasis. A trend of increase in the transcript level of adenosine kinase (ADK) was also found, but did not reach statistical significance. Importantly, J4 treatment normalized all these changes (Table 1), suggesting that J4 was able to reinstate proper adenosine homeostasis in the hippocampus of Tau22 mice.

**Table 1:** Enzymes involved in adenosine homeostasis

Gene	WTC	WTJ	TauC	TauJ
*ADA*	1.06 ± 0.09	1.04 ± 0.06	3.47 ± 0.42*	1.35 ± 0.24^#^
*ADK*	1.02 ± 0.03	1.06 ± 0.02	1.17 ± 0.06	0.85 ± 0.09^#^
*CD39*	1.01 ± 0.03	1.23 ± 0.02*	1.45 ± 0.06*	0.94 ± 0.05^#^
*CD73*	1.02 ± 0.04	1.23 ± 0.07*	1.33 ± 0.05*	1.13 ± 0.05^#^

Mice were treated as indicated (control WT mice, WTC; J4-treated WT mice, WTJ; control Tau22 mice, TauC; and J4-treated Tau22 mice, TauJ; n = 6–9 in each group) from the age of 3–11 months. The hippocampus was harvested carefully and subjected to RT-qPCR. GAPDH was used as a reference gene for normalization. The data are expressed as the mean ± SEM. *p < 0.05 versus the WT vehicle group; ^#^*p* < 0.05 versus the Tau22 vehicle group

### Chronic J4 treatment prevents impairment of spatial learning and memory of Tau22 mice

**Fig. 1:**
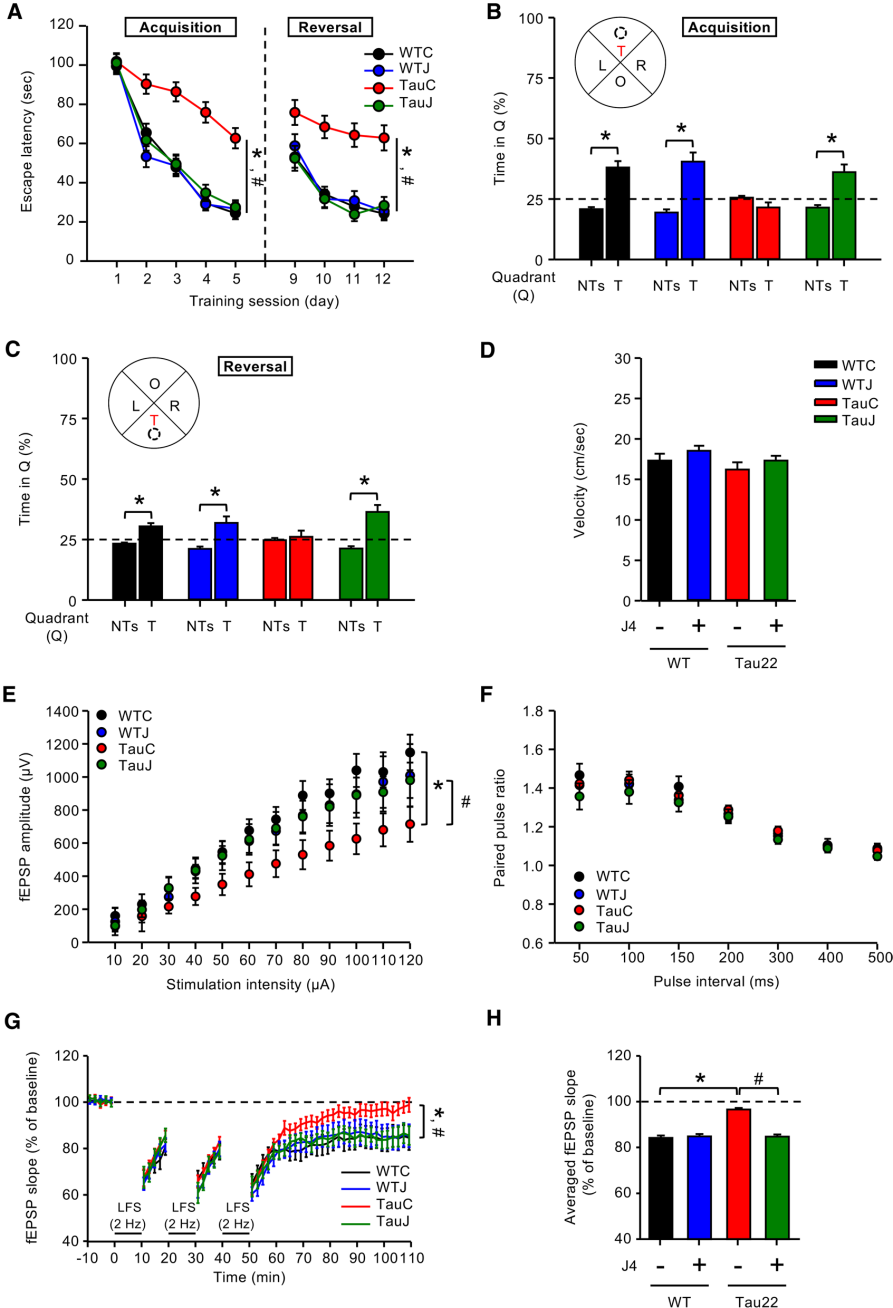
Chronic treatment with J4 alleviates the impairment of spatial memory and hippocampal CA1 LTD in Tau22 mice. **a–d** Mice were treated as indicated (WTC, black; WTJ, blue; TauC, red; TauJ, green; n = 12–20 in each group) from the age of 3–10 months. The Morris water maze (MWM) with a hidden platform was used to assess spatial learning and memory. **a** The acquisition-learning phase (Day 1–Day 5) and the reversal-learning phase (Day 9–Day 12) of MWM. *p < 0.05 versus the WTC group; ^#^*p* < 0.05 versus the TauC group; two-way ANOVA. Probe tests for **b** the spatial memory and **c** spatial reversal memory were performed on Day 8 and Day 15, respectively. The average percentage of time spent in the T (target quadrant) and NTs (nontarget quadrants) were calculated. *p < 0.05 versus the NTs; two-tailed Student’s *t-*test. **d** The swimming speed (cm/s) of the animals in the probe test. **e–h** Hippocampal slices were prepared from mice subjected to different treatment groups (n = 3–18 slices from 3 to 8 mice in each group) from the age of 3–10 months. **e** The input/output relationship curve (fEPSP responses, stimuli strengths increased from 10 to 120 μA), **f** averaged paired-pulse ratios (interstimulus intervals, from 50 to 500 ms), and **g**, **h** Long-term depression (LTD) induced by 3 trains of LFS (2 Hz, 1200 pulses) at the Schaffer collateral-CA1 synapses were recorded. **g** The average fEPSP slopes of mice subjected to different treatments were calculated. *p < 0.05 versus the WTC group; ^#^*p* < 0.05 versus the TauC group, two-way ANOVA. **h** Quantification results of the mean fEPSP slopes during the last 10 min of the steady-state period. *p < 0.05 versus the WTC group; ^#^*p* < 0.05 versus the TauC group, one-way ANOVA. The data are expressed as means ± S.E.M

Under the tested condition, the spatial memory and cognitive flexibility of mice at the age of 10 months were evaluated using the Morris water maze (MWM) task. J4 significantly prevented spatial learning deficits in vehicle-treated Tau22 mice (TauC) during the acquisition-learning phase (Day 1–Day 5) (Fig. 1a) without affecting wild-type mice. Moreover, vehicle-, J4-treated WT, and J4-treated Tau22, but not vehicle-treated Tau22 mice showed a preference for searching the hidden platform in the target quadrant (T) over the nontarget quadrants (NTs) in the probe test (Fig. 1b), indicating that J4 improved the impaired memory of Tau22 mice.

To assess cognitive flexibility, we also examined the spatial reversal learning of WT and Tau22 mice treated with or without J4 for an additional four consecutive days. In the reversal-learning phase (Day 9–Day 12), the hidden platform was relocated to the opposite quadrant and the escape latency was recorded. J4 improved the impaired spatial reversal learning of TauC mice (Fig. 1a). Animals were subjected to the probe test on Day 15. Except for vehicle-treated Tau22 mice, all mice spent more time in the new target quadrant (T) than in the nontarget quadrants (NTs) (Fig. 1c). No difference in the swimming speed was observed among the groups tested (Fig. 1d). Together, J4 significantly improves the spatial memory and spatial reversal memory of Tau22 mice.

### Chronic J4 treatment normalizes the impairment of hippocampal CA1 LTD in Tau22 mice

We next investigated whether J4 affects the synaptic plasticity in Tau22 mice. The basal transmission of the hippocampal CA3–CA1 network was determined based on the input–output relationship. The vehicle-treated Tau22 mice showed decreased basal synaptic transmission compared to that of the vehicle-treated WT mice. Such abnormal synaptic transmission was alleviated by J4 in Tau22 mice (Fig. 1e). Presynaptic neurotransmitter release in the hippocampus, as determined by the paired-pulse facilitation (PPF) response assay, was comparable between genotypes (Tau22 versus WT mice) and treatment groups (J4 versus vehicle) (Fig. 1f), suggesting that presynaptic plasticity was unaffected in Tau22 mice.

Previous studies have demonstrated that Tau22 mice exhibit impaired long-term depression (LTD) but normal long-term potentiation (LTP) at the Schaffer collateral-CA1 synapses in the hippocampus [33]. As shown in Fig. 1g, LTD was maintained in WTC mice but not in TauC mice. This impairment in LTD was prevented by J4. The average LTD magnitude during the last 10 min of recording was quantified and shown in Fig. 1h. Collectively, J4 normalizes the impaired basal synaptic transmission and LTD at Schaffer collateral synapses in Tau22 hippocampi without affecting those in WT hippocampi.

### Chronic J4 treatment reduces Tau hyperphosphorylation in the hippocampi of Tau22 mice

**Fig. 2:**
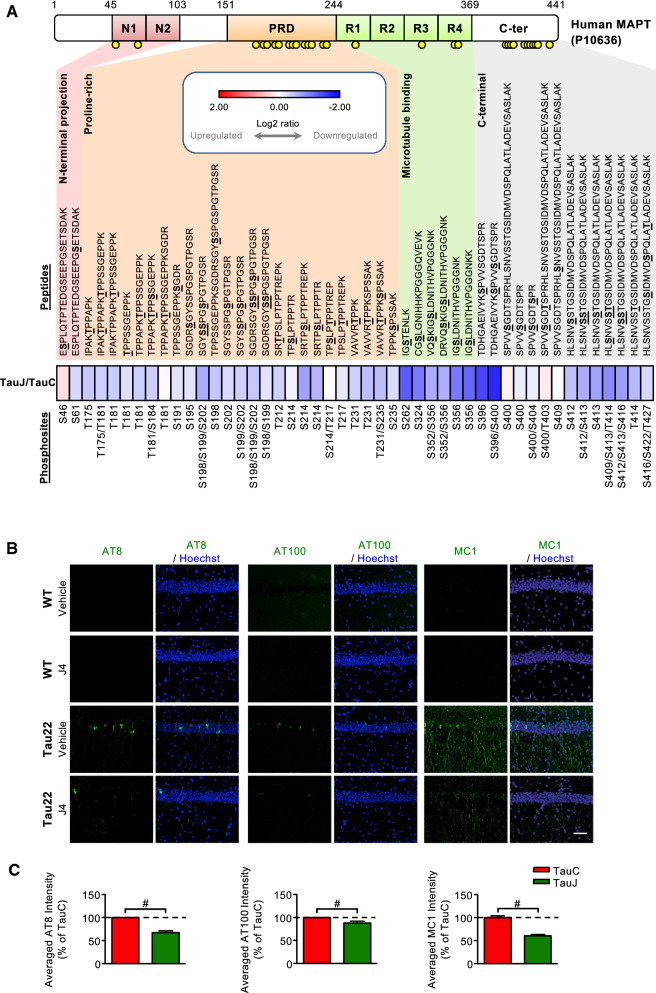
Chronic J4 treatment decreases hyperphosphorylated and misfolded human Tau levels in the hippocampi of Tau22 mice. Mice were treated as indicated (WTC, black; WTJ, blue; TauC, red; TauJ, green) for 8–9 months from the age of 3 months. **a** Pooled total hippocampal lysates (200 μg) from 3 animals of the age of 12 months were harvested and subjected to phospho-proteomic analysis. The heatmap shows the relative log2 expression ratio of phosphorylated human tau (MAPT, P10636) in the TauJ group vs. the TauC group. The relative expression level (log2 ratio) of human phosphorylated tau is shown on a scale from red (upregulated) to blue (downregulated). The identified phosphorylation sites on peptides are shown in bold and underlined. **b**, **c** Hippocampal sections (20 μm) were prepared from mice with different treatment groups (n = 3–5 in each group) from the age of 3–11 months and subjected to IHC staining. **b** The levels of hyperphosphorylated tau and misfolded tau in the hippocampus were evaluated by staining with the indicated antibodies (AT8 for Ser202/Thr205, green; AT100 for Thr212/Ser214, green; MC1 for conformational changed tau, green), and the quantification results are shown in (**c**). Scale bar, 50 μm. The data are expressed as the mean ± S.E.M. ^#^*p* < 0.05, versus the TauC group, two-tailed Student’s *t-*test

To examine the effect of J4 on Tau phosphorylation [33], we performed a differential peptide labeling (iTRAQ) of hippocampal proteins and analyzed the results using LC–MS/MS-based proteomics. Phosphopeptides covering 30 phosphorylation sites of human Tau were identified (Fig. 2a). In total, J4 reduced phosphorylation at 15 phosphorylation sites. The J4-mediated reduction in Tau phosphorylation at sites commonly observed in Alzheimer’s Disease was further assessed by Western blot and immunofluorescence analysis using antibodies raised against hyperphosphorylated (pThr181, pSer199, pSer202/Thr205 = AT8, pSer262, pSer396, and pSer422) and misfolded (pThr212/Ser214 = AT100; MC1) Tau. Chronic treatment with J4 significantly reduced Tau phosphorylation at the sites tested except for pSer181 (Figs. 2b, c, Additional file 1: S3A, and S3B). No effect of J4 on the human Tau was observed, demonstrating that J4 did not modulate the Thy-1 promoter directly (Additional file 1: Fig. S3C–S3G). Importantly, the levels of misfolded Tau, assessed by AT100 and MC1 [44, 45], were also reduced by J4 (Fig. 2b, c). Collectively, J4 reduces the levels of hyperphosphorylated and misfolded Tau in Tau22 hippocampi.

### Chronic J4 treatment reduces the AMPK activation in the hippocampus of Tau22 mice

We next examined the phosphorylation level of 17 kinases and signaling molecules by using the MAPK Phosphorylation Array (RayBiotech, GA, USA). Only minor or no change was found between Tau22 mice and WT mice (Fig. S4). No marked alterations in the level or activation of PP2A [46], the major tau phosphatase, were detected either (Additional file 1: Fig. S5).

**Fig. 3:**
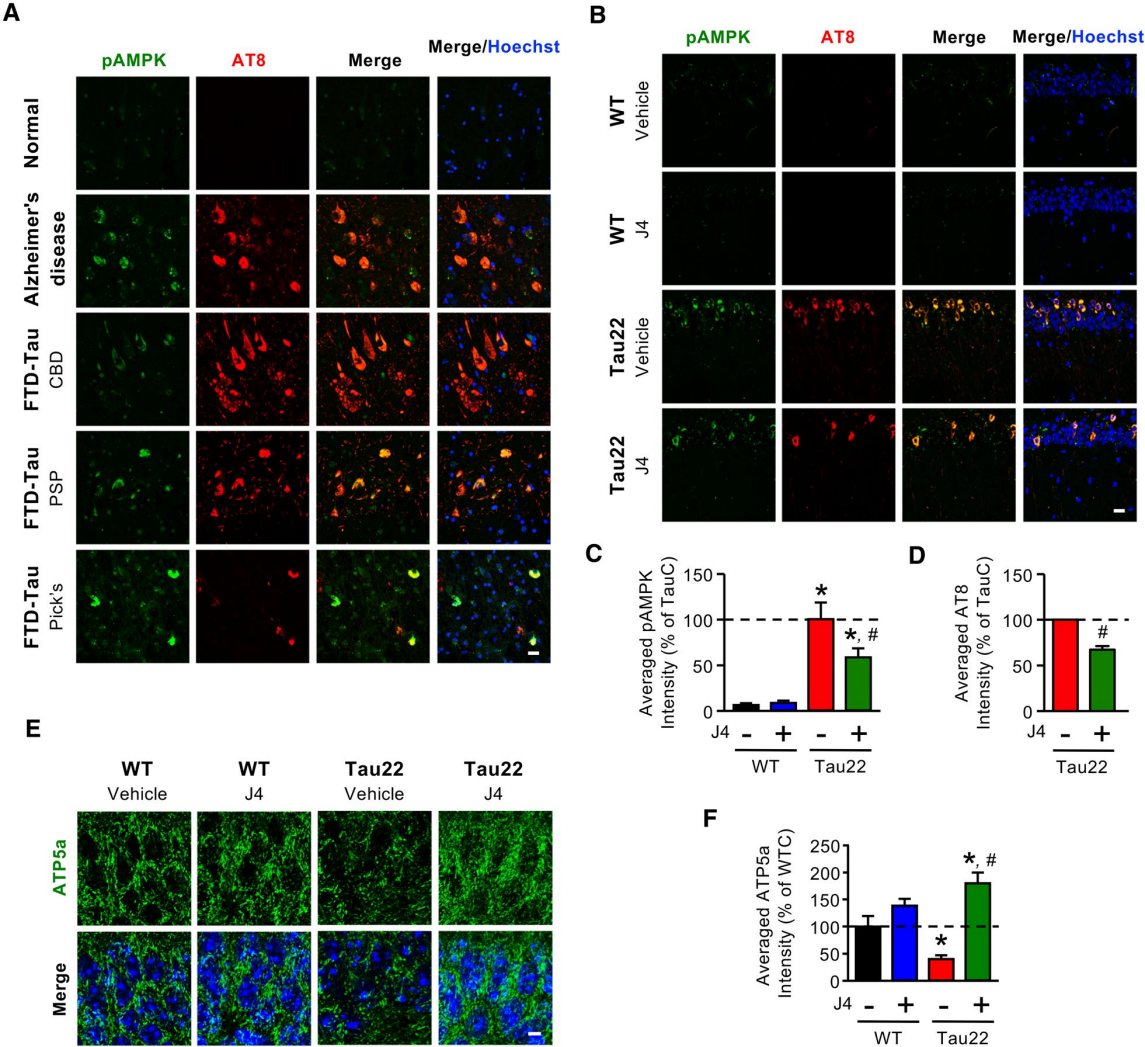
Chronic J4 treatment decreases AMPK activation and rescues mitochondrial abnormalities in the hippocampi of Tau22 mice. **a** Posterior hippocampal sections (6 μm) from normal subjects and Alzheimer’s Disease and FTD-Tau (CBD, PSP, and Pick’s disease) patients were subjected to IHC staining. The levels of phospho-AMPK and hyperphosphorylated tau were evaluated by staining with the indicated antibodies (pAMPK^Thr172^, green; AT8 for pTau^Ser202/Thr205^, red). **b–f** Mice were treated as indicated (WTC, black; WTJ, blue; TauC, red; TauJ, green; n = 5–7 in each group) from the age of 3–11 months. Hippocampal sections (20 μm) were prepared and subjected to IHC staining using the indicated antibodies (pAMPK^Thr172^, green; AT8 for pTau^Ser202/Thr205^, red), and the staining was quantified (**c**, **d**). Scale bar, 20 μm. **e**, **f** The level of the mitochondrial marker ATP5a was evaluated by staining with an anti-ATP5a antibody (**e**, green) and quantified (**f**; n = 3 in each group). Scale bar, 5 μm. The data are expressed as the mean ± S.E.M. *p < 0.05 versus the WTC group; ^#^*p* < 0.05, versus the TauC group, one-way ANOVA

Because adenosine homeostasis has been implicated in the regulation of AMPK activation [31, 32, 47] and since AMPK directly phosphorylates Tau [48], we evaluated the activation/phosphorylation of AMPK (Thr^172^, designated pAMPK) and Tau phosphorylation in the hippocampi of postmortem Alzheimer’s Disease, FTLD-Tau patients, and Tau22 mice. Immunofluorescence staining revealed that, in the posterior hippocampal sections from Alzheimer’s Disease and FTD-Tau patients, pAMPK was detected in neurons that contained phosphorylated Tau (Figs. 3a and Additional file 1: S6). Similarly, elevated AMPK phosphorylation was observed in neurons containing phosphorylated Tau in the CA1 region in Tau22 mice, but rarely in those in WT mice (Fig. 3b, c). J4 treatment decreased the levels of pAMPK and pTau in Tau22 mice compared to WT mice (Fig. 3b–d). In line with this finding, analyses of hippocampal proteins using a combination of iTRAQ and LC–MS/MS-based proteomics by Phosphopeptides revealed that the phosphorylation levels of two AMPK downstream targets (i.e., eukaryotic elongation factor 2 (eEF2) and myosin VI (Myo6); [49, 50]) and one upstream regulator (Ca^2+^/calmodulin-dependent protein kinase kinase-β, Camk2b; [51]) were elevated in Tau22 mice. J4 treatment reduced the phosphorylation of eEF2, Myo6, and Camk2b (Additional file 1: Table S6), supporting that J4 normalized the upregulated AMPK signaling pathway in the hippocampus of Tau22 mice.

Since AMPK is a critical energy sensor and Tau has been implicated in mitochondrial dysfunction [52], we next assessed mitochondrial mass by immunohistochemical staining using an antibody against ATP5a, a component of complex V [53]. Consistent with abnormal AMPK activation, TauC mice exhibited less ATP5a-positive mitochondrial mass in the hippocampus than WTC mice (Fig. 3e). J4 reversed mitochondrial loss in Tau22 mice (Fig. 3e, f) as it normalized the AMPK overactivation (Fig. 3b, c), suggesting that the blockade of ENT1 normalized Tau-associated energy dysfunction.

### Transcriptomic signature associated with the beneficial effect of J4 in the hippocampi of Tau22 mice

**Fig. 4:**
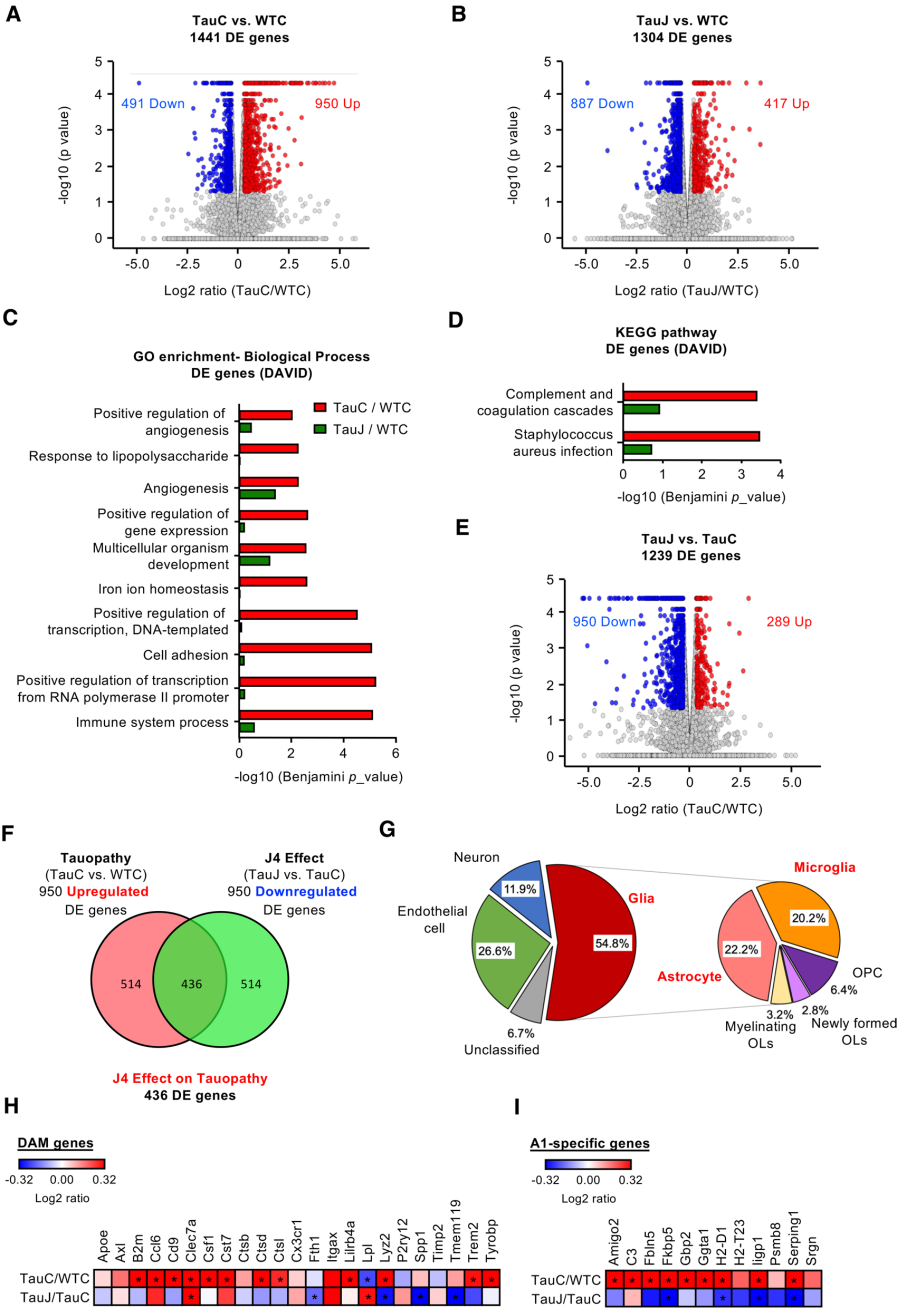
Chronic J4 treatment ameliorates the expression of tauopathy associated genes and neurotoxic reactive astrocyte genes in the hippocampi of Tau22 mice. Mice were treated as indicated (WTC, TauC, and TauJ; n = 3 in each group) from the age of 3–10 months. The hippocampus was carefully removed for RNA-seq analysis. Volcano plot of DE genes in the **a** TauC group vs. WTC group and **b** TauJ group vs. WTC group. **c** GO enrichment analysis of DE genes between the TauC and WTC groups (red bar) and TauJ and WTC groups (green bar). **d** KEGG pathway analysis of DE genes between the TauC and WTC groups (red bar) and TauJ and WTC groups (green bar). **e** Volcano plot of the DE genes in the TauJ group vs. TauC group. In the volcano plot, the significantly upregulated and downregulated DE genes (absolute log_2_ ratio ≥ 0.32; *p* < 0.05) are shown in red and blue, respectively. **f** Venn diagram showing the 436 overlapping DE genes between the upregulated DE genes in the TauC group vs. WTC group (pink) and the downregulated DE genes in the TauJ group vs. TauC group (green). **g** Pie chart of cell-type-enrichment of 436 tauopathy-associated DE genes regulated by J4. In the volcano plot, the significantly upregulated and downregulated DE genes (absolute log_2_ ratio ≥ 0.32; *p* < 0.05) are shown in red and blue, respectively. In the Venn diagram and pie chart, the number and percentage of DE genes in each category were shown in each sector. **h**, **i** Heatmap of **h** DAM genes and **i** A1-specific genes in the TauC/WTC and TauJ/TauC groups. The relative expression level (log_2_ ratio) of genes is shown on a scale from red (upregulated) to blue (downregulated). Asterisks indicate significant alterations (*p* < 0.05)

To gain mechanistic insight into J4’s action, we performed transcriptional profiling of the hippocampus using RNA-seq analysis. A total of 1441 differentially expressed (DE) genes (950 upregulated and 491 downregulated) between TauC and WTC mice were identified (Fig. 4a, TauC mice versus WTC mice, *p* < 0.05 and absolute log_2_ fold-change ≥ 0.32). Between the TauJ and WTC groups, a total of 1304 DE genes were also identified (Fig. 4b; TauJ mice versus WTC mice, *p* < 0.05, absolute log_2_ fold-change ≥ 0.32) but the proportion of upregulated (417) and downregulated (887) markedly differed. Interestingly, only a fraction (216 upregulated; 214 downregulated; (Additional file 1: Fig. S7A and S7D) of the genes affected in Tau mice as compared to WT, remained altered in the Tau mice after J4 treatment. These data suggested that J4 significantly normalized the expressions of Tauopathy-associated genes. Gene ontology (GO; Fig. 4c, FDR < 0.05, Benjamini *p* < 0.01) and Kyoto Encyclopedia of Genes and Genomes (KEGG; Fig. 4d, FDR < 0.05, Benjamini *p* < 0.01) analyses were conducted, and the DE genes between the TauC group vs WTC group were found to be associated with the immune response and transcriptional machinery. Most of the pathways deregulated by Tau pathology (red bars in Fig. 4c, d) became less significant after J4 treatment (green bars). When we analyzed the upregulation- or downregulation-specific DE genes separately (Additional file 1: Fig. S7A–S7C and S7D–S7F, respectively), multiple pathways (e.g., transcription-related machineries, angiogenesis, cell–cell interaction, cell adhesion) remained markedly normalized by J4 treatment. Part of the inflammation-related pathways were also rescued by J4 treatment.

Next, we specifically analyzed the effect of J4 on Tau22 mice vs. control Tau22 mice. A total of 1239 DE genes were identified (289 upregulated and 950 downregulated; TauJ mice versus TauC mice, *p* < 0.05 and absolute log_2_ fold-change ≥ 0.32; Fig. 4e). Importantly, J4 normalized the expression of 436 of the 950 upregulated DE genes (45.8%; Fig. 4f) and 85 of the 491 downregulated DE genes (17.3%, data not shown) between the TauC group and WTC group. Moreover, J4 normalized multiple dysregulated canonical pathways, which were also observed in other Alzheimer's disease mouse models (Tg4510 and APP/PS1), in Tau22 mice (Additional file 1: Fig. S8). Overall, J4 had a broad impact on Alzheimer’s Disease-related signaling molecules and pathways in Tau22 hippocampi.

### Chronic J4 treatment mitigates the activation of microglia in the hippocampi of Tau22 mice

**Fig. 5:**
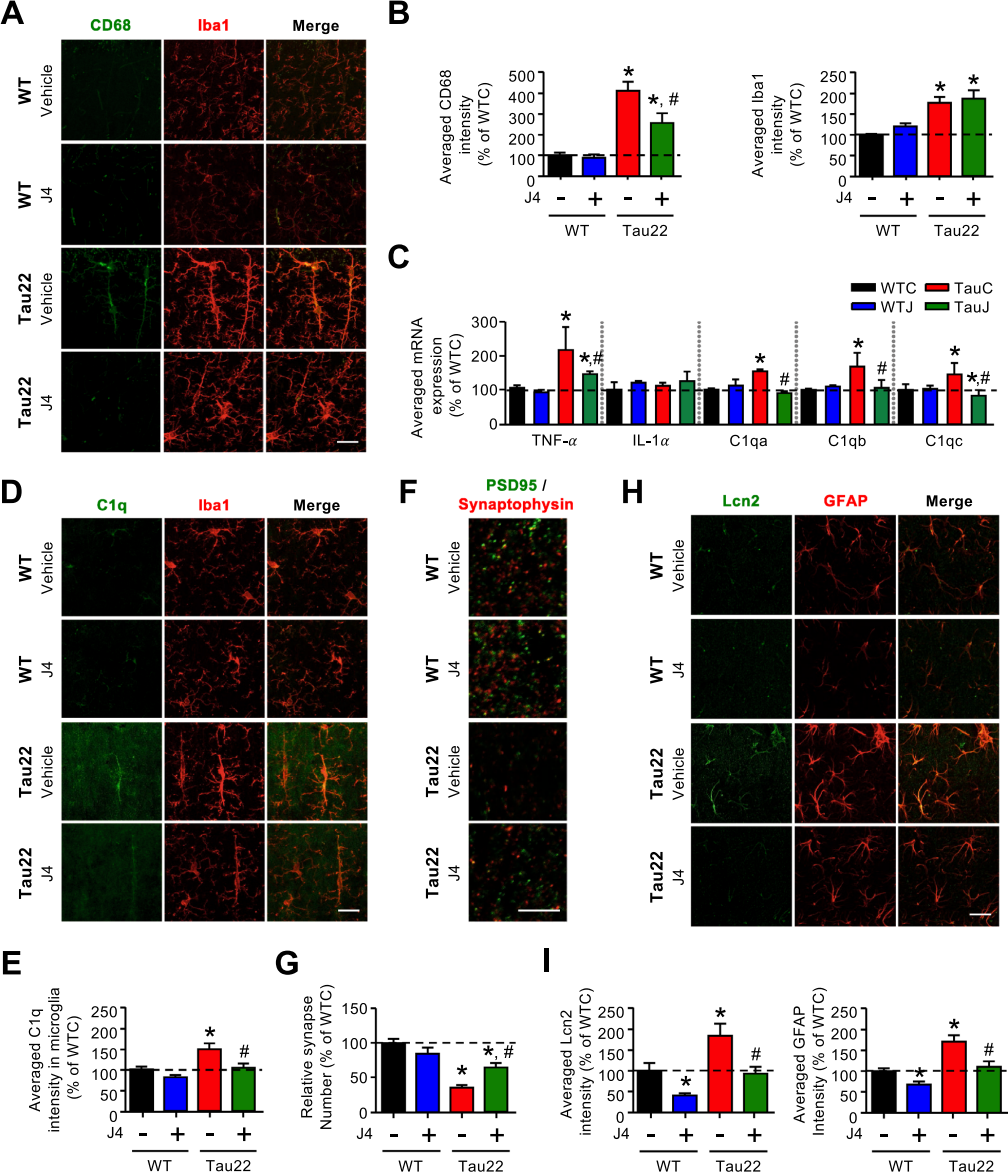
Chronic J4 treatment suppresses the activation of microglia and the induction of cytotoxic A1 astrocytes in the hippocampi of Tau22 mice. Mice were treated as indicated (WTC, black; WTJ, blue; TauC, red; TauJ, green; n = 5–7 in each group) from the age of 3–11 months, and their tissues were subjected to IHC staining and RT-qPCR analysis. **a** CD68 (green) is a marker of reactive microglia and Iba1 (red) is a marker of microglia and the quantification results are shown in (**b**). **c** Gene expression of TNF-α, IL-1α, and C1q (C1qa, C1qb, and C1qc) in the hippocampi of treated mice (n = 6–9 in each group) was analyzed, and GAPDH was used as a reference gene. **d** The intensity of C1q (green) and Iba1 (red) expression was examined and the quantification results are shown in (**e**). Scale bar, 20 μm. **f**, **g** The number of synapses (**f**) in the hippocampi of treated mice (n = 10–12 in each group) was evaluated by staining with the indicated antibodies (PSD95, green; SYP, red). The quantification results are shown in (**g**). Scale bar, 5 μm. **h** Hippocampal sections (20 μm) were prepared and subjected to IHC staining (Lcn2 a marker of reactive astrocytes, green; GFAP a marker of astrocytes, red) and the staining was quantified (**i**). Scale bar, 20 μm. The data are expressed as the mean ± S.E.M. *p < 0.05 versus the WTC group; ^#^*p* < 0.05 versus the TauC group, one-way ANOVA

Based on the cell-type information listed in the Brain RNAseq database (https://www.brainrnaseq.org, [54]), we further classified the 436 DE genes with expression levels normalized by J4 (Fig. 4f) into five types (including neuron-enriched, glia-enriched, endothelial cell-enriched, and unclassified). As shown in Fig. 4g, approximately 55% of the DE genes whose expression levels that were normalized by J4 were enriched in glial cells, including astrocytes and microglia (Fig. 4g). In Tau22 hippocampi, enhanced gene signatures for the disease-associated microglia were detected (DAM, [55]; Fig. 4h and Additional file 1: Table S7). Consistently, the immunoreactive intensities of both Iba1 (a marker of microglia) and CD68 (a marker of activated microglia) were significantly elevated in the hippocampi of TauC mice (11 months old) compared with the hippocampi of WTC mice (Fig. 5a, b). We also measured the transcript levels of several factors secreted by activated microglia and known to favor neurotoxic activation of astrocytes (A1 phenotype; [56]) using RT-qPCR. As shown in Fig. 5c, the levels of *TNF-α* and *C1q* (*C1qa*, *C1qb*, and *C1qc*), but not *IL-1α*, were upregulated in the Tau22 hippocampi compared with WT hippocampi. Immunofluorescence staining and RT-qPCR showed that J4 prevented the upregulation of *TNF-α*, C1q, and CD68 in Tau22 mice (Fig. 5a–e, Additional file 1: S9A and S9B), suggesting that J4 mitigated microglial inflammation in Tau22 mice.

### Chronic J4 treatment mitigates synaptic loss in Tau22 mice

C1q is an important mediator of Tau-induced synaptic loss [34, 57]. We thus examined the number of synapses by immunofluorescence staining. In Tau22 hippocampi, the levels of a postsynaptic marker (PSD95) and a presynaptic marker (synaptophysin) were lower than those in WT hippocampi. Consistent with the rescuing effect of J4 on C1q, J4 restored the levels of PSD95 and synaptophysin in Tau22 mice (Additional file 1: Fig. S9C and S9D). We also determined the number of synapses based on the colocalization of PSD95 and synaptophysin. In line with the reduction in CD68 and C1q levels (Fig. 5a–c), J4 rescued the synaptic loss in Tau22 hippocampi [58] (Fig. 5f, g).

### Chronic J4 treatment suppresses the cytotoxic astrocytes induction in the hippocampi of Tau22 mice

TNF-α and C1q are potent astrocytic activators for the neurotoxic A1 phenotype [56]. Our RNA-seq analysis of Tau22 hippocampi revealed the upregulation of gene signatures of pan-reactive and cytotoxic A1 astrocytes ([59], Additional file 1: Table S8 and Fig. 4i). Immunofluorescence staining further showed that Tau22 mice (TauC) exhibited higher levels of GFAP (an astrocyte marker) and Lcn2 (a pan reactive astrocyte marker, [60]) in their hippocampus than WT mice (WTC; Fig. 5h, i), confirming that astrocytes in Tau22 hippocampi were abnormally activated. Interestingly, FTD-Tau patients and late stage Alzheimer’s Disease patients (Additional file 1: Fig. S10), upregulation of several A1-specific genes (e.g., *GBP2*, *SERPING1*, *FKBP5*) was observed. Notably, the five A1 astrocyte genes tested were all significantly upregulated in the frontal cortex of FTD-Tau-Pick’s disease patients compared to control subjects, suggesting a link between Tau and astrocyte reactivity (Additional file 1: Fig. S10). The induction of reactive A1 astrocytes appears secondary to microglial activation in Tau22 hippocampi since activated microglia (CD68-positive) were detected in the hippocampi of young Tau22 mice (4 months old), when no reactive astrocytes (Lcn2-positive) were observed (Additional file 1: Fig. S11).

According to the reduction of the microglial phenotype as well as *TNFα* and *C1q* expressions (Fig. 5c), chronic J4 treatment normalized not only GFAP and Lcn2 levels but also the pathological upregulation of A1-specific genes expression (Figs. 4i, 5h, i; Additional file 1: Table S8). Collectively, our data suggest that early Tau-induced microglial activation is likely to promote the activation of neurotoxic astrocytes and can be blocked by J4.

## Discussion

The present study showed that chronic treatment with J4, an ENT1 blocker, mitigates Tau pathology by alleviating not only mitochondrial dysfunction and AMPK overactivation but also the neuroinflammatory status of microglia and astroglia, ultimately attenuating the impairment of compromised synapses as well as spatial learning and memory. Our study particularly supports a functional link between adenosine homeostasis, AMPK regulation and Tau pathology development.

Mitochondrial dysfunction is a major pathogenic feature of Alzheimer’s Disease [61] and is known to facilitate the hyperphosphorylation of Tau, which in turn alters the morphology and functions of mitochondria [62]. Therefore, it is not surprising that AMPK, a key energy sensor and an upstream kinase of Tau, is overactivated in the hippocampi of patients with Alzheimer’s Disease or tauopathies [28]. One major function of AMPK is the maintenance of cellular energy homeostasis through modulation of the balance between anabolic and catabolic processes [29]. Because hippocampal neurons of WT mice are homeostatic in nature, no significant AMPK activation was observed in the hippocampus of WT mice (Fig. 3b, c). Treatment with J4 showed no impact on AMPK activation in such a homeostatic condition, suggesting that ENT1 does not play a significant role in the regulation of AMPK in physiological conditions. Conversely, the impaired energy status of hippocampal neurons of Tau22 mice (i.e., in an allostatic situation) causes the activation of AMPK. We hypothesized that blockade of ENT1 may reduce the entry of adenosine and, subsequently the cellular level of AMP, thereby altering the AMP/ATP ratio, and ultimately suppressing AMPK activation in hippocampal neurons of Tau22 mice. Our hypothesis is in line with a recent study demonstrating that genetic deletion of ENT1 in erythrocytes reduces adenosine uptake and leads to the suppression of AMPK [47].

Accumulating evidence demonstrates that overactivation of AMPK in neurons causes synapse loss via an autophagy-dependent pathway, and links synaptic integrity and energetic failure in neurodegenerative diseases [63]. Here, we found that aberrant AMPK activation was associated with synaptic loss and reduced basal synaptic transmission in Tau22 hippocampi (Figs. 1e, 3b, c, 5f, g; Additional file 1: Table S6). Collectively, J4 suppresses AMPK overactivation, and normalizes impaired neuronal plasticity in both APP/PS1 and Tau22 mice (LTP and LTD, respectively; [27]; Fig. 1g, h).

Besides the impaired cognitive function, we did not observe any obvious systemic alteration of Tau22 up to 12 months except for a slightly lower body weight. Treatment with J4 did not affect the bodyweight of Tau22 and WT mice (Additional file 1: Fig. S12), suggesting that chronic J4 treatment at the condition tested had no obvious toxicity. Although significant Tau hyperphosphorylation and gliosis were observed in the hippocampus of Tau22 mice of 10–12 months (Figs. 2 and 5), no change in the volume of the whole brain, hippocampus, and ventricle were altered (Additional file 1: Fig. S13). Because J4 is a blocker of ENT1, we measured the levels of adenosine in the hippocampus of Tau22 mice (11 months old) but found no difference in either the extracellular or the intracellular steady state levels by in vivo microdialysis and tissue extraction coupled to high performance liquid chromatography (HPLC), respectively (Figs. S1 and S14). This is probably because what we measured were bulk adenosine concentrations in the extracellular fluid and those inside of cells in the hippocampus. To assess whether adenosine levels are altered in microenvironments (e.g., extracellular space proximal to neuronal soma and synapses), future investigations using an in vivo adenosine sensor [64] will be needed. Nonetheless, the levels of at least three genes (i.e., ADA, CD39, CD73) involved in adenosine metabolism were elevated in the hippocampus of Tau22 mice. A trend of increase in the transcript level of ADK was also found, but did not reach statistical significance. Importantly, J4 treatment normalized all these changes (Table 1), suggesting that adenosine homeostasis was altered in Tau22 mice. It is possible that the elevation of ADA in the hippocampus of Tau22 mice may reduce adenosine availability from intracellular source, while the elevation of CD39 and CD73 increases extracellular adenosine pool, which counteracts the imbalance of intracellular adenosine level. This may be why the adenosine alteration was not observed in the hippocampus of Tau22 mice (Additional file 1: Fig. S14). J4 treatment reset adenosine homeostasis by blockading adenosine entry, which results in the decrease of ADA, CD39, and CD73 in the hippocampus of Tau22 mice. Collectively, chronic J4 does not induce a major change in the steady state level of adenosine but rather adenosine homeostasis.

An interesting study recently reported that Tau22 mice are more susceptible to pentylenetetrazol (PTZ) for seizure and mortality than WT mice, probably due to the enhanced expression of ADK [65]. This is of great interest because J4 is an anti-epileptic agent in a PTZ-induced kindling model [66]. Given that J4 treatment reduced the level of ADK in Tau22 mice (Table 1), it is plausible that modulation of ADK may contribute to the beneficial effect of J4 in Tau22 mice. No effect of J4 on the expressions of A_1_ adenosine receptor, A_2A_ adenosine receptor and some of their signaling molecules (i.e., protein kinase A, GSK3β, AMPKs) were observed (Additional file 1: Table S9).

Although pathogenic Tau is specifically expressed in the neurons of mouse models of tauopathy (including Tau22 and rTg4510 mice), reactive microglia and astrocytes have been found near neurons that contain high levels of pathogenic Tau, suggesting that degenerating neurons may trigger abnormal gliosis [33] and alter their gene expression profiles [67]. Here, our RNA-seq analysis of Tau22 mice showed upregulation of several genes associated with the disease-associated microglia (DAM, [55]) in mice and patients with Alzheimer’s Disease (Fig. 4h and Additional file 1: Table S7). Although J4 did not rescue all inflammation-related pathways as analyzed by GO and KEGG (Additional file 1: Fig. S7), 56 of the 90 dysregulated homeostatic microglia genes (62%; [68]) of Tau22 mice were normalized by J4 treatment (Fig. S15). These homeostatic microglial genes are commonly expressed in microglia of healthy adult brain. Thus, J4 treatment rescued the dysregulated microglial homeostasis in Tauopathy.

Notably, J4 not only ameliorated the energy dysfunction and Tau pathology in neurons (Figs. 1, 2, 3) but also markedly reduced the activation of CD68-positive microglia activation and rescued the synapse loss associated with the phagocytic activities of activated microglia (Fig. 5) [34, 57, 69]. Given that CD68 is also a marker of phagocytosis [70], the suppression of CD68 by J4 suggest that J4 treatment might decrease the phagocytic capacity of microglia and result in the rescue of synapse loss (Fig. 5b), in agreement with the normalization of C1q expression by J4, a microglial complement protein known to regulate synaptic phagocytosis by microglia in a Tau pathology context [34, 69]. We also performed RT-qPCR and established that microglial factors known to promote neurotoxic activation of astrocytes were upregulated in Tau22 mice (*C1q* and *TNFα*; Fig. 5c). Consistent with the suppression of microglial activation, J4 also significantly reduced astrocytic activation, with a particular impact on the A1 signature (Figs. 4i, 5h, i). We show, for the first time, a pathological activation of the A1 neurotoxic astrocytic phenotype in a tauopathy context using both human and mouse samples (Additional file 1: Fig. S10 and Table S8), which may be related to the production of A1-promoting factors by microglia. Our RNA-seq analysis of Tau22 mice also emphasizes the upregulation of a disease-associated astrocytes (DAAs) signature, recently detected in an amyloid Alzheimer’s Disease mouse model (5XFAD) [71] (Additional file 1: Table S10). These observations are of particular importance since glial activation has been shown to be instrumental in Alzheimer’s Disease [5]. In accordance, the beneficial effects of J4 on memory alterations and neuroplasticity (Figs. 1, 2, 3) is associated with the normalization of both A1-promoting factors and A1 astrocytes in Tau22 mice (Additional file 1: Table S8). Treatment with J4 normalized 60% of the elevated DAA genes in Tau22 mice (Additional file 1: Table S10), further supporting the beneficial effect of J4 on the prevention of abnormal astrocytic activation. Noteworthy, ENT1 is not only expressed by neurons but also by astrocytes. Earlier studies had reported that blockade of ENT1 in astrocytes suppresses the expression of two astrocyte-specific genes (i.e., the type 2 excitatory amino acid transporter (EAAT2) and aquaporin 4 (AQP4)) [72]. Because J4 treatment did not affect the level of EAAT2 and AQP4 (Additional file 1: Table S9), suppression of astrocytic ENT1 by J4 under the condition tested was unlikely to be significant.

## Conclusions

In summary, we provide evidence that an ENT1 inhibitor (J4) rescues the energy dysfunction (including mitochondrial impairment and AMPK overactivation) and pathological glial activation, and subsequently improves synaptic function and memory in tauopathy. Modulation of adenosine homeostasis by an ENT1 inhibitor (J4) therefore deserves further development in tauopathies and Alzheimer’s Disease.

## Supplementary Information

**Additional file 1**. Supplementary Information.

## Data Availability

The data that support the findings of this study are available on request to the corresponding author.

## Abbreviations

Aβ: Amyloid-beta
ACN: Acetonitrile
ACSF: Artificial cerebrospinal fluid
AD: Alzheimer’s disease
ADA: Adenosine deaminase
ADK: Adenosine kinase
AMPK: AMP-activated protein kinase
BBB: Blood–brain barrier
BSA: Bovine serum albumin
CBD: Corticobasal degeneration
cDNA: Complementary DNA
DAAs: Disease-associated astrocytes
DAM: Disease-associated microglia
DAVID: Database for Annotation, Visualization and Integrated Discovery
DE: Differentially expressed
DTT: Dithiothreitol
ENT1: Equilibrative nucleoside transporter 1
FDR: False discovery rate
fEPSPs: Field excitatory postsynaptic potentials
FPKM: Fragments per kilobase per million
FTD: Frontotemporal dementia
GO: Gene Ontology
HPβCD: 2-Hydroxypropyl-b-cyclodextrin
IAA: Iodoacetamide
IACUC: Institutional Animal Care and Utilization Committee
IVC: In ventilated cages
KEGG: Kyoto Encyclopedia of Genes and Genomes
LFS: Low-frequency stimulation
LTD: Long-term depression
LTP: Long-term potentiation
MWM: Morris water maze
NDS: Normal donkey serum
NGS: Normal goat serum
PCF: Proteomics Core Facility
PP2A: Protein phosphatase 2A
PPF: Paired-pulse facilitation
PSP: Progressive supranuclear palsy
RT-qPCR: Reverse Transcription quantitative PCR
PTZ: Pentylenetetrazol
SBS: Sequencing-by-synthesis
Tau22: THY-Tau22
TauC: Vehicle-treated Tau22 mice
TauJ: J4-treated Tau22
TEAB: Triethylammonium bicarbonate
TFA: Trifluoroacetic acid
WT: Wild type
WTC: Vehicle-treated WT mice
WTJ: J4-treated WT
