# Supplementary material for: Equilibrative nucleoside transporter 1 inhibition rescues energy dysfunction and pathology in a model of tauopathy
Source: Acta Neuropathol Commun. 2021 Jun 22;9:112. doi: 10.1186/s40478-021-01213-7 (PMC8220833; doi:10.1186/s40478-021-01213-7)
Supplement: Supplementary file 1 — Additional file 1. Supplementary Information. [file 40478_2021_1213_MOESM1_ESM.pdf]

Full title: **Equilibrative nucleoside transporter 1 inhibition rescues energy dysfunction and pathology in a model of tauopathy**

Authors: Ching-Pang Chang[1], Ya-Gin Chang[1], Pei-Yun Chuang[1], Thi Ngoc Anh Nguyen[1], Kuo-Chen Wu[2], Fang-Yi Chou[2], Sin-Jhong Cheng[1,3], Hui-Mei Chen[1], Lee-Way Jin[4], Kevin Carvalho[5,6], Vincent Huin[5,6], Luc Buée[5,6], Yung-Feng Liao[7], Chun-Jung Lin[2], David Blum[5,6], Yijuang Chern[1]

Affiliations: [1]Institute of Biomedical Sciences, Academia Sinica, Taipei, Taiwan; [2]School of Pharmacy, National Taiwan University, Taipei, Taiwan; [3]Neuroscience Program of Academia Sinica, Academia Sinica, Taipei, Taiwan; [4]Department of Pathology and Laboratory Medicine, University of California Davis, Sacramento, CA, USA; [5]Univ. Lille, Inserm, CHU Lille, U1172 - LilNCog - Lille Neuroscience & Cognition, F-59000 Lille, France; [6]Alzheimer & Tauopathies, LabEx DISTALZ, LiCEND, F-59000 Lille, France; [7]Institute of Cellular and Organismic Biology, Academia Sinica, Taipei, Taiwan

Corresponding author:

Dr. Yijuang Chern; Institute of Biomedical Sciences, Academia Sinica, Nankang, Taipei 115, Taiwan; Tel: 886-2-26523913; Fax: 886-2-27829143; E-mail: bmychern@ibms.sinica.edu.tw

Dr. David Blum; Univ. Lille, Inserm, CHU Lille, U1172 - LilNCog - Lille Neuroscience & Cognition, F-59000 Lille, France; Tel: +33320298858; E-mail: david.blum@inserm.fr

Inventory of Supplemental Information

a) Supplemental Experimental Procedures

b) Supplemental Tables

c) Supplemental Figures and Legends

d) Supplemental References

Supplemental Experimental Procedures

Bioavailability and brain distribution of J4

Mice were administered 10 mg/kg of J4 via oral gavage or 1 mg/kg of J4 intravenously. Aliquots of blood samples were collected at 2, 10, and 30 mins and 1, 2, 4, 6, and 8 hrs after dosing post-dose. After protein precipitation with acetonitrile, the blood samples were stored at -80°C until analysis.

For brain distribution of J4, mice were treated with J4 intravenously. At each predetermined time point (2, 15, and 30 mins and 1, 2, 4, 6, and 8 hrs after dosing), blood and brain were collected. The blood samples were processed as described above. The whole brain was carefully removed from the skull and homogenized with 100% methanol using a glass homogenizer. After being centrifuged, the supernatant was transferred to a glass tube and evaporated to dryness with a gentle stream of nitrogen. The samples were reconstituted in the mobile phase and then centrifuged for further analysis.

The concentrations of J4 in mouse blood and brain were determined by liquid chromatography coupled with tandem mass spectrometry (LC-MS/MS) as described previously [3]. The area under the concentration-time curve from time zero to the time of the last quantifiable concentration (AUC_{0-t}) was calculated using the trapezoidal rule. The absolute bioavailability was calculated by the ratio of dose-normalized average AUC values of oral and intravenous administration. The brain-to-blood ratio was generated from the AUC values of the brain and blood.

Measurements of adenosine level in the brain

For *in vivo* brain microdialysis, mice were anesthetized with ketamine (90 mg/kg) and xylazine (10 mg/kg) via intraperitoneal injection followed by placing its head in a fixed position on a stereotaxic instrument (Stoelting, Wood Dale, IL, USA). The vertical guide cannula was implanted into the hippocampus at specific coordinates (anteroposterior, 2.8 mm; mediolateral, 3.0 mm from Bregma; and dorsoventral, 2.3 mm). The mice were allowed to rest for 24 hrs. A microdialysis probe (MAB 10.8.2.Cu, Microbiotech/se AB, Stockholm, Swedish) was inserted into the brain through the guide cannula. Mice were transferred to an isolated cage and infused (flow rate of 1.0 μL/min) with the Ringer's solution (2.3 mM $CaCl_2$, 4 mM KCl, and 147 mM NaCl) through the microdialysis probe for 4 hrs, followed by infusing the mouse with J4 (1 mM in 2% DMSO containing Ringer's solution) for another 4 hrs. The brain outflow was collected every 30 mins and stored at -20 °C until derivatization.

For brain extraction, mice were rapidly decapitated and the hippocampus was dissected out and homogenized with 85 μl of ice-cold 0.6 N perchloric acids followed by centrifuging at 11,000 x g for 30 mins at 4 °C. The supernatant was collected, neutralized with 2 M K_2CO_3, vortexed for 30 sec followed by centrifuging at 11,000 x g for 10 mins at 4 °C. The upper aqueous layer was collected and stored at 80 °C until derivatization.

To measure the adenosine level, the collected microdialysis sample or brain extracts were converted into fluorescent 1, N^6-etheno-adenine derivatives as previously described [1]. Briefly, the samples were mixed with the freshly prepared reaction buffer (7.8 M chloroacetaldehyde and 1 M acetate buffer, pH 4.5 in an

11.2:138.8 ratio by volume) at a 1:3 ratio. The samples were centrifuged at 500 x g for 2 mins at RT and then heated at 60 °C for 60 mins. The reaction was terminated by placing samples on ice. After centrifugation at 500 x g for 2 min, the etheno-derivatives were analyzed by high-performance liquid chromatography (HPLC). Chromatographic separation was performed on a Kinetex 5μ C18 100A column (150 x 4.6 mm, Phenomenex Inc., Torrance, CA, USA) equipped with a C18 SecurityGuard cartridge (Phenomenex). For microdialysis samples, the elution was carried out in a gradient program with a mobile phase composed of buffer A (30 mM KH_2PO_4 and 0.8 mM tetra-n-butylammonium bromide, pH 5.45) and buffer B [acetonitrile/30 mM KH_2PO_4 (1:1, by volume) and 0.8 mM tetra-n-butylammonium bromide, pH 7.0)] at a flow rate of 1 ml/min. For brain extracts, the derivatized adenosine was eluted off the column at a flow rate of 0.8 ml/min by a mobile phase (0.1 M KH_2PO_4 and 5 mM tetra-n-butylammonium bromide in 2% ACN, pH 3.3) with an isocratic manner. The etheno-derivatives were detected by a fluorescence detector (L2200, Hitachi, Tokyo, Japan) using an excitation wavelength of 280 nm and an emission wavelength of 410 nm.

Phospho-kinase array

Mouse hippocampal lysates collected from three animals from each condition were analyzed with the Human/Mouse MAPK Phosphorylation Array (C-Series, Cat# AAH-MAPK-1-4, RayBiotech Inc., USA) according to the manufacturer's protocol. The samples (1 mg/ml in the blocking buffer) were loaded onto a membrane that preincubated with the blocking buffer for 30 mins at RT and incubated overnight at 4 °C. The membrane was then washed with wash buffer I and wash buffer II for 3 times each and then incubated with a detection antibody cocktail overnight at 4 °C. After extensive washes, the membrane was incubated with HRP-conjugated anti-rabbit IgG overnight at 4 °C and washed extensively. The immunosignals were monitored using a chemiluminescent detection buffer. The intensity of each spot on the membrane was quantified with ImageJ software (NIH, Bethesda, MD, USA) and normalized to the intensities of the positive control on the same membrane. The relative kinase activity was assessed by normalizing the signal of the phosphorylated substrate to that of the positive controls.

Immunoblotting

After the animals were rapidly decapitated, the hippocampus was dissected out, homogenized in an ice-cold detergent-free lysis buffer (10 mM Tris-HCl, pH 7.4, and 0.32 M sucrose) containing inhibitors of protease (50X complete EDTA free Tab, Roche Diagnostics, IN, USA) and phosphatase inhibitors (10x PhosSTOP, Roche Diagnostics) using sterilized tissue grinder (FocusBio, Taiwan) and then passed through an insulin syringe 15 times. The tissue lysates were then centrifuged at 800 x g for 10 mins at 4 °C to remove the debris. The supernatants were collected and stored at -80 °C until use. The protein concentration was measured using the Bio-Rad Protein Assay Dye Reagent Concentrate (Bio-Rad, Hercules, CA, USA). For Western blot analyses, total proteins (20 μg) were first separated via SDS-PAGE (10%-12%), transferred onto PVDF membranes (0.45 μm pore size, Millipore, MA, USA), hybridized with the indicated primary antibody (Table S5) overnight at 4 °C, and then incubated with the corresponding horseradish peroxidase (HRP)-conjugated

secondary antibody for 1 hr at RT. After extensive washes, the immunosignals were detected using enhanced chemiluminescence (ECL) reagent (PerkinElmer, MA, USA).

Morphological analysis of brain

The brain structures of the mice were measured using micro-magnetic resonance imaging (MRI) analysis in the Animal Imaging Facility (AIF). Brain tissues were collected from normal saline perfused mice. The isolated brain tissues were embedded in 1.5% agarose, followed by subjecting to ex vivo micro-MRI imaging. Images of the overall brain structure, hippocampus, and ventricles were evaluated using a Bruker Biospec 7 Tesla MRI scanner (Bruker, Germany). All images and volumes were processed and measured using Amira (Thermo Fisher Scientific, Waltham, MA, USA). The brain mass (mm^3) was calculated by subtracting ventricle volume from total brain volume.

Supplemental Tables

Supplemental Table 1. Bioavailability and brain distribution of J4 in mice

	Mean ± S.E.M.
**The bioavailability estimation**	
AUC_{blood} (ng*h/mL) (i.v. 1 mg/kg)	1421.8 ± 358.9
AUC_{blood} (ng*h/mL) (oral 10 mg/kg)	6778.9 ± 651.0
**F (%)**	**48**
**The brain-to-blood ratio examination**	
AUC_{brain} (ng*h/mL)	159.9 ± 7.4
AUC_{blood} (ng*h/mL)	994.4 ± 112.0
**Brain-to-blood ratio (%)**	**16.4 ± 1.6**

For bioavailability estimation, mice (n=3) were administered with J4 through a single intravenous injection (1 mg/kg) or oral gavage (10 mg/kg). For the brain-to-blood ratio examination, mice (n=3) were administered with J4 through a single intravenous injection (1 mg/kg). The data are expressed as the means ± S.E.M. AUC: area under the concentration-time curve, which represents the systemic/brain exposure of a drug after the drug is administered; AUC_{blood}: area under the blood concentration-time curve from time zero to the time of the last measurable concentration; AUC_{brain}: area under the brain concentration-time curve from time zero to the time of the last measurable concentration; F: bioavailability, which is defined as the fraction of the drug in the systemic circulation when orally administered and is estimated by the ratio of AUC_{blood} following oral administration to that following intravenous administration, normalized by the doses.

Supplemental Table 2. Human cases analyzed in this study

DX	NA ID	Braak	Age	Sex	PMI (hrs)	Region	Application
Alzheimer’s disease	4543	6	71	M	9	Hp	IF
Alzheimer’s disease	4552	6	64	F	6	Hp	IF
Alzheimer’s disease	4580	6	73	M	12	Hp	IF
Alzheimer’s disease	4851	6	69	M	17	Hp	IF
Alzheimer’s disease	4872	NA	71	F	NA	Hp	IF
Alzheimer’s disease	4966	6	75	F	17	Hp	IF
FTD-Tau (CBD)	4855	5	72	F	19.5	Hp	IF
FTD-Tau (Pick's)	4439	0	69	M	12	Hp	IF
FTD-Tau (Pick's)	4798	0	74	F	18	Hp	IF
FTD-Tau (Pick's)	4814	0	68	F	24	Hp	IF
FTD-Tau (Pick's)	4933	2	74	M	19	Hp	IF
FTD-Tau (PSP)	4744	1	65	F	8	Hp	IF
Normal	4706	1	75	F	3.5	Hp	IF
Normal	4727	1	78	F	NA	Hp	IF
Normal	4768	1	82	F	14.17	Hp	IF
Normal	4795	2	75	M	18	Hp	IF
Normal	4858	2	78	F	40.83	Hp	IF
Normal	4894	2	75	M	49.5	Hp	IF
Alzheimer’s disease /MCI	C09-19424	4	88	F	16	BA10	RT-qPCR
Alzheimer’s disease /MCI	G 56	4	90	F	4	BA10	RT-qPCR
Alzheimer’s disease /MCI	G 73	3	92	F	4	BA10	RT-qPCR
Alzheimer’s disease /MCI	G 89	4	68	M	6	BA10	RT-qPCR
Alzheimer’s disease /MCI	P 7024	4	76	F	28	BA10	RT-qPCR
Alzheimer’s disease	C07-20298	6	77	F	15	BA10	RT-qPCR
Alzheimer’s disease	C07-20991	6	80	F	5	BA10	RT-qPCR
Alzheimer’s disease	C08-03086	6	86	M	10	BA10	RT-qPCR
Alzheimer’s disease	C08-10048	5	62	M	10	BA10	RT-qPCR
Alzheimer’s disease	C08-28113	6	86	F	8	BA10	RT-qPCR
Alzheimer’s disease	C08-31992	5	89	F	24	BA10	RT-qPCR

Alzheimer’s disease	C09-34184	6	73	F	22	BA10	RT-qPCR
Alzheimer’s disease	C10-03400	6	72	F	5.5	BA10	RT-qPCR
Alzheimer’s disease	C11-34515	6	67	M	9.5	BA10	RT-qPCR
Alzheimer’s disease	C11-36501	6	65	M	36	BA10	RT-qPCR
Alzheimer’s disease	C12-00612	6	61	F	17	BA10	RT-qPCR
Alzheimer’s disease	C12-05247	6	83	F	3	BA10	RT-qPCR
Alzheimer’s disease	C12-10400	5	89	H	20	BA10	RT-qPCR
Alzheimer’s disease	G 109	5	92	F	NA	BA10	RT-qPCR
FTD-Tau (CBD)	C08-09824	NA	75	F	7	BA10	RT-qPCR
FTD-Tau (CBD)	C08-18677	NA	76	M	19	BA10	RT-qPCR
FTD-Tau (CBD)	C08-29472	NA	71	M	5	BA10	RT-qPCR
FTD-Tau (CBD)	P 5925	NA	72	F	15	BA10	RT-qPCR
FTD-Tau (CBD)	P 8671	NA	81	F	33	BA10	RT-qPCR
FTD-Tau (PSP)	C08-04599	NA	77	F	17	BA10	RT-qPCR
FTD-Tau (PSP)	C08-18602	3	75	M	7	BA10	RT-qPCR
FTD-Tau (PSP)	C09-17736	NA	65	M	18	BA10	RT-qPCR
FTD-Tau (PSP)	C10-06503	NA	82	F	11	BA10	RT-qPCR
FTD-Tau (PSP)	C10-07949	NA	77	F	24	BA10	RT-qPCR
FTD-Tau (PSP)	C12-37132	NA	77	F	16	BA10	RT-qPCR
FTD-Tau (PSP)	C14-00229	NA	89	M	36	BA10	RT-qPCR
FTD-Tau (PSP)	P 3814	NA	76	M	7	BA10	RT-qPCR
FTD-Tau (PSP)	P 4117	NA	70	M	NA	BA10	RT-qPCR
FTD-Tau (PSP)	P 7622	NA	80	F	22	BA10	RT-qPCR
FTD-Tau (Pick's)	C09-21706	NA	71	M	21	BA10	RT-qPCR
FTD-Tau (Pick's)	C10-05402	NA	68	F	11	BA10	RT-qPCR
FTD-Tau (Pick's)	C11-33155	NA	68	M	15	BA10	RT-qPCR
FTD-Tau (Pick's)	P 3538	NA	60	M	NA	BA10	RT-qPCR
FTD-Tau (Pick's)	P 8245	NA	67	F	NA	BA10	RT-qPCR
Normal	C06-14473	2	72	M	18	BA10	RT-qPCR
Normal	C08-05295	0	52	F	28	BA10	RT-qPCR
Normal	P 3549	2	69	M	6	BA10	RT-qPCR
Normal	P 3862	1-2	58	M	5.5	BA10	RT-qPCR
Normal	P 4078	2	73	M	10	BA10	RT-qPCR
Normal	P 8730	1-2	82	M	NA	BA10	RT-qPCR
Normal	P 8866	1-2	84	M	15.5	BA10	RT-qPCR
Normal	G 15	1	80	M	26	BA10	RT-qPCR
Normal	G 18	1	76	F	8	BA10	RT-qPCR
Normal	G 69	1	95	F	3	BA10	RT-qPCR

FTD-Tau (P301L)	A02 1102	NA	43	M	6	TpCx	RT-qPCR
FTD-Tau (P301L)	A02 1213	NA	66	M	30	TpCx	RT-qPCR
FTD-Tau (P301L)	A06 2025	NA	65	F	NA	TpCx	RT-qPCR
Normal	A09 0606	NA	70	M	30	TpCx	RT-qPCR
Normal	A11 1620	NA	72	M	7	TpCx	RT-qPCR
Normal	A14 0019	NA	53	F	29	TpCx	RT-qPCR

Alzheimer's disease/MCI, Mild cognitive impairment with early Alzheimer's disease diagnosis; FTD, Frontotemporal dementia; CBD, Corticobasal degeneration; PSP, Progressive supranuclear palsy; PMI, Postmortem interval; M, Male; F, Female; Hp, Posterior hippocampus; BA10, Prefrontal cortex Brodmann area 10; TpCx, Temporal Cortex; NA, not available; IF, immunofluorescence; RT-qPCR, quantitative reverse transcription PCR.

Supplemental Table 3. Sequences of the RT-qPCR primers used in this study

Gene name	Primer sequence		Product size (bp)
mAda	Forward	5’-AACATTATCGGCATGGACAAGC-3’	132 bp
	Reverse	5’-TGCCTTCATCTCCACAAACTCG-3’	
mAdk	Forward	5’-GTGCTATTTGGAATGGGGAAT-3’	207 bp
	Reverse	5’-CAACCACTGAGCCACTTTCAT-3’	
mCD39 (Entpd1)	Forward	5’-GTACCTGAGTGAGTACTGCTTCTC-3’	165 bp
	Reverse	5’-GCTGGGATGTTGGTCAAGTTC-3’	
mCD73 (Nt5e)	Forward	5’-CAAATCCCACACAACCACTG-3’	158 bp
	Reverse	5’-TGCTCACTTGGTCACAGGAC-3’	
mC1qa	Forward	5’-GAGCACCCAACGGGAAGGAT-3’	111 bp
	Reverse	5’-GGATACCAGTCCGGATGCCA-3’	
mC1qb	Forward	5’-CACAGAACACCAGGATTCCATAC-3’	109 bp
	Reverse	5’-GGAGAAAACCTAGAAGCAGCAGT-3’	
mC1qc	Forward	5’-GCGATGAGGTGTGGCTATCA-3’	174 bp
	Reverse	5’-GGAAGAGGTCTGAGTGAGGATG-3’	
mGAPDH	Forward	5’-TGACATCAAGAAGGTGGTGAAG-3’	109 bp
	Reverse	5’-AGAGTGGGAGTTGCTGTTGAAG-3’	
mIL1-α	Forward	5’-TGCAGTCCATAACCCATGATC-3’	113 bp
	Reverse	5’-GACAAACTTCTGCCTGACGAG-3’	
mTNFα	Forward	5’-TCTTCTCATTCCTGCTTGTGG-3’	130 bp
	Reverse	5’-AGGGTCTGGGCCATAGAACT-3’	

The sequences of the forward and reverse primers (5'→3' direction) for each gene of interest used for RT-qPCR assays are listed.

Supplemental Table 4. The list of TaqMan probes used in this study

Assay ID	Gene	Target species	Assay design	Amplicon length (bp)
Hs00163811_m1	C3	Human	Probe spans exons	88 bp
Hs00163781_m1	SERPING1	Human	Probe spans exons	70 bp
Hs01561006_m1	FKBP5	Human	Probe spans exons	75 bp
Hs01056636_m1	FBLN5	Human	Probe spans exons	65 bp
Hs00894837_m1	GBP2	Human	Probe spans exons	84 bp
Hs99999903_m1	ACTB	Human	Amplicon spans exons	171 bp

The TaqMan assay ID and design for each gene of interest used for RT-qPCR assays are listed.

Supplemental Table 5. List of antibodies

Name	Abbreviation	Source	Species reactivity	Dilution	Source (Cat No.)
Anti-Glial fibrillary acidic protein	GFAP	Rabbit	Mouse, Human	IF: 1:500	Sigma-Aldrich (G9269)
Anti-Glial fibrillary acidic protein	GFAP	Mouse	Mouse, Human	IF: 1:200	Sigma-Aldrich (G3893)
Anti-Iba1	Iba1	Rabbit	Mouse, Human	IF: 1:500	Wako (019-19741)
AIF-1/Iba1	Iba1	Goat	Mouse, Human	IF: 1:300	Novus Biologicals (NB100-1028)
Lipocalin-2/ NGAL	Lcn2	Goat	Mouse	IF: 1:200	R&D (AF1857)
Anti-CD68 (ED1)	CD68	Mouse	Mouse, Human	IF: 1:100	Abcam (ab31630)
Phospho-Tau (Ser199)	p-Tau^{Ser199}	Rabbit	Mouse, Human	WB: 1:1000	Thermo Fisher Scientific (44-734G)
Phospho-Tau (Thr212, Ser214)	AT100	Mouse	Human	IF: 1:1000<br>WB: 1:1000	Thermo Fisher Scientific (MN1060)
Phospho-Tau (Ser202, Thr205)	AT8	Mouse	Mouse, Human	IF: 1:2000	Thermo Fisher Scientific (MN1020)
Phospho-Tau (Ser262)	p-Tau^{Ser262}	Rabbit	Mouse, Human	WB: 1:1000	Thermo Fisher Scientific (44-750G)
Phospho-Tau (Ser396)	p-Tau^{Ser396}	Rabbit	Mouse, Human	WB: 1:2000	Thermo Fisher Scientific (44-752G)
Phospho-Tau (Ser422)	p-Tau^{Ser422}	Rabbit	Mouse, Human	WB: 1:1000	Thermo Fisher Scientific (44-764G)
Phospho-Tau (Thr181)	AT270	Mouse	Human	WB: 1:1000	Thermo Fisher Scientific (MN1050)
Tau (MC1)	MC1	Mouse	Mouse, Human	IF: 1:1000<br>WB: 1:1000	Gift from Dr. Peter Davies
Phospho-Tau (Ser396, Ser404) (PHF1)	PHF1	Mouse	Human	WB: 1:1000	Gift from Dr. Peter Davies
Anti-Tau	Tau-5	Mouse	Mouse, Human	WB: 1:2500	Thermo Fisher Scientific (AHB0042)
Anti-Human Tau	HT7	Mouse	Human	WB: 1:2000	Thermo Fisher Scientific (MN1000)
Phospho-AMPKα (Thr172)	p-AMPK^{Thr172}	Rabbit	Mouse, Human	IF: 1:50	Cell Signaling (#2535)
Anti-ATP5A antibody [15H4C4]	ATP5A	Mouse	Mouse, Human	IF: 1:200	Abcam (ab14748)
Anti-C1q antibody [4.8]	C1q	Rabbit	Mouse	IF: 750	Abcam (ab227072)
Anti-PSD95, clone 7E3-1B8	PSD95	Mouse	Mouse, Human	IF: 1:250	Millipore (MAB1598)
SYP (C-20)	SYP	Goat	Mouse, Human	IF: 1:100	Santa Cruz (Sc-7568)

Supplemental Table 6. J4 treatment normalizes the aberrant AMPK signaling in the hippocampus of Tau22 mice.

Gene Symbol	Phosphorylation Site	TauC/WTC	TauJ/TauC	Reference
Camk2b	T287	0.86	-0.29	[5]
eEF2	T57	0.96	-0.20	[2]
Myo6	T406	1.02	-0.54	[4]

Pooled total hippocampal lysates (200 μg) from 3 animals at the age of 12 months were harvested and subjected to phospho-proteomic analysis. The phosphorylation level of indicated proteins (relative log2 ratio) in the TauC group vs. the WTC group or TauJ group vs. the TauC group are shown. Camk2b, Ca^{2+}/calmodulin-dependent protein kinase kinase-β; eEF2, eukaryotic elongation factor 2; Myo6, myosin VI.

Supplemental Table 7. Disease-associated microglia (DAM) genes

Type	Gene name	TauC/WTC	*p*_value	TauJ/TauC	*p*_value
**DAM genes**	*Apoe*	0.05	0.62	-0.07	0.54
	Axl	0.18	0.10	0.04	0.73
	Ccl6	**0.69***	0.00	0.27	0.14
	Cd9	**0.58***	0.00	-0.12	0.15
	Clec7a	**2.91***	0.00	**0.68***	0.01
	Csf1	**0.37***	0.00	-0.01	0.89
	Cst7	**2.80***	0.00	0.24	0.31
	Ctsb	0.12	0.14	-0.06	0.44
	Ctsd	**0.41***	0.00	-0.13	0.10
	Ctsl	**0.27***	0.00	-0.10	0.29
	Cx3cr1	0.06	0.69	0.11	0.45
	Fth1	-0.04	0.63	**-0.19**#	0.03
	Itgax	3.24	1.00	0.34	0.37
	Lilrb4a	**1.54***	0.00	0.02	0.96
	Lpl	**-0.23***	0.03	**0.30***	0.01
	Lyz2	**1.06***	0.00	**-0.30**#	0.01
	P2ry12	-0.11	0.43	0.13	0.38
	Spp1	0.08	0.58	**-0.49**#	0.00
	Timp2	-0.08	0.30	0.06	0.42
	Tmem119	0.12	0.21	**-0.32**#	0.00
	Trem2	**0.30***	0.04	-0.23	0.15
	Tyrobp	**0.33***	0.02	-0.02	0.90

Mice were treated as indicated conditions (control WT mice, WTC; control Tau22 mice, TauC; and J4-treated Tau22 mice, TauJ; n= 3 in each group) from the age of 3-10 months. The hippocampus was harvested carefully and subjected to RNA-seq analysis. The relative levels (log2 ratio) of DAM genes are listed. *$p<0.05$ versus WTC mice; #$p<0.05$ versus TauC mice.

Supplemental Table 8. PAN-reactive and A1-specific genes

Type	Gene name	TauC/WTC	*p*_value	TauJ/TauC	*p*_value
**PAN-reactive genes**	*Aspg*	**0.81***	0.01	-0.25	0.40
	Cd44	0.27	0.08	**0.31#**	0.04
	Cp	0.28	0.09	-0.18	0.24
	Cxcl10	0.60	0.29	0.48	0.37
	Gfap	**0.73***	0.00	-0.14	0.22
	Hsbp1	-0.12	0.14	-0.04	0.60
	Lcn2	3.44	0.28	-2.40	0.15
	Osmr	**0.56***	0.00	0.00	0.98
	S1pr3	0.07	0.59	0.02	0.89
	Serpina3n	**0.52***	0.00	0.03	0.72
	Steap4	**1.58***	0.00	**-1.53#**	0.00
	Timp1	1.16	1.00	-0.12	1.00
	Vim	**0.37***	0.00	**-0.22#**	0.03
**A1-specific genes**	*Amigo2*	**0.63***	0.00	-0.16	0.10
	C3	**1.33***	0.00	0.09	0.83
	Fbln5	**0.61***	0.00	-0.29	0.11
	Fkbp5	**0.42***	0.00	**-0.38#**	0.00
	Gbp2	**0.84***	0.00	-0.07	0.66
	Ggta1	**0.68***	0.00	-0.13	0.40
	H2-D1	**0.56***	0.00	**-0.26#**	0.01
	H2-T23	0.20	0.30	-0.25	0.18
	Iigp1	**0.85***	0.00	**-0.41#**	0.02
	Psmb8	0.09	0.74	-0.26	0.35
	Serping1	**0.46***	0.00	**-0.49#**	0.00
	Srgn	0.19	0.16	-0.12	0.38

Mice were treated as indicated conditions (control WT mice, WTC; control Tau22 mice, TauC; and J4-treated Tau22 mice, TauJ; n= 3 in each group) from the age of 3-10 months. The hippocampus was harvested carefully and subjected to RNA-seq analysis. The data are expressed as the log2 ratio. *p<0.05 versus the WT vehicle group; #p<0.05 versus the Tau22 vehicle group.

Supplemental Table 9. Gene expression level of adenosine-related proteins

Gene name	TauC/WTC	*p*_value	TauJ/TauC	*p*_value
Adora1 (A_1R)	0.04	0.72	0.05	0.65
Adora2a ($A_{2A}R$)	0.06	0.84	0.16	0.60
Prkaa1 (AMPKα1)	0.19	0.17	0.02	0.90
Prkaa2 (AMPKα2)	0.01	0.94	0.08	0.39
Prkaca (PKA)	0.06	0.46	-0.12	0.14
Slc29a1 (ENT1)	0.05	0.72	0.11	0.46
Slc1a2 (EAAT2)	0.08	0.48	0.07	0.53
Aqp4	0.15	0.05	0.07	0.35

Mice were treated as indicated conditions (control WT mice, WTC; control Tau22 mice, TauC; and J4-treated Tau22 mice, TauJ; n= 3 in each group) from the age of 3 to 10 months. The hippocampus was harvested carefully and subjected to RNA-seq analysis. The data are expressed as the log2 ratio. *$p<0.05$ versus the WT vehicle group; #$p<0.05$ versus the Tau22 vehicle group.

Supplemental Table 10. **The upregulated DAAs genes in the hippocampus of Tau22 mice**

Type	Gene name	TauC/WTC	*p*_value	TauJ/TauC	*p*_value
**DAA/GFAP low** (38/239 upregulated genes)	*Abca1*	**0.45***	0.00	-0.02	0.86
	Aspg	**0.81***	0.01	-0.25	0.40
	B2m	**0.33***	0.00	-0.09	0.27
	C1qa	**0.45***	0.00	**-0.21**$^{\#}$	0.01
	C4b	**1.16***	0.00	0.00	0.99
	Cd151	**0.40***	0.00	**-0.54**$^{\#}$	0.00
	Cd9	**0.58***	0.00	-0.12	0.15
	Ctsd	**0.41***	0.00	-0.13	0.10
	Ezr	**0.44***	0.00	**-0.37**$^{\#}$	0.00
	Fxyd1	**0.59***	0.00	**-0.54**$^{\#}$	0.00
	Gadd45g	**0.34***	0.00	**-0.40**$^{\#}$	0.00
	Gfap	**0.73***	0.00	-0.14	0.22
	Ggta1	**0.68***	0.00	-0.13	0.40
	H2-D1	**0.56***	0.00	**-0.26**$^{\#}$	0.01
	H2-K1	**0.53***	0.00	**-0.34**$^{\#}$	0.00
	Id4	**0.42***	0.00	**-0.43**$^{\#}$	0.00
	Lamp2	**0.37***	0.00	**-0.19**$^{\#}$	0.03
	Lgals3bp	**0.92***	0.00	**-0.39**$^{\#}$	0.00
	Mgst1	**0.49***	0.00	**-0.40**$^{\#}$	0.00
	Mt1	**0.57***	0.00	**-0.67**$^{\#}$	0.00
	Mt2	**0.81***	0.00	**-0.72**$^{\#}$	0.00
	Nfe2l2	**0.57***	0.00	**-0.34**$^{\#}$	0.00
	Nfkbia	**0.96***	0.00	**-0.85**$^{\#}$	0.00
	Osmr	**0.56***	0.00	0.00	0.98
	Pard3b	**0.48***	0.00	-0.13	0.35
	Pdgfd	**0.48***	0.00	**-0.31**$^{\#}$	0.03
	Pdzd2	**0.40***	0.02	**-0.39**$^{\#}$	0.03
	Plce1	**0.72***	0.00	-0.15	0.31
	Sdc4	**0.66***	0.00	**-0.33**$^{\#}$	0.00
	Serpina3n	**0.52***	0.00	0.03	0.72
	Serpinf1	**0.51***	0.00	**-0.44**$^{\#}$	0.01
	Serping1	**0.46***	0.00	**-0.49**$^{\#}$	0.00
	Slc14a1	**0.36***	0.00	0.10	0.28
	Stat3	**0.37***	0.00	-0.06	0.58
	Sulf1	**1.97***	0.00	**-1.66**$^{\#}$	0.00
	Thbs4	**0.40***	0.00	-0.16	0.12
	Usp53	**0.49***	0.00	**-0.31**$^{\#}$	0.00
	Vim	**0.37***	0.00	**-0.22**$^{\#}$	0.03

The data are expressed as the log2 ratio. *p<0.05 versus the WT vehicle group; $^{\#}p$<0.05 versus the Tau22 vehicle group.

Supplemental Table 10. The upregulated DAAs genes in the hippocampus of Tau22 mice (Continued)

Type	Gene name	TauC/WTC	*p*_value	TauJ/TauC	*p*_value
**GFAP high/GFAP low** (31/311 upregulated genes)	*Acsl3*	**0.36***	0.01	-0.04	0.79
	Ass1	**0.35***	0.00	**-0.33#**	0.00
	B2m	**0.33***	0.00	-0.09	0.27
	C4b	**1.16***	0.00	0.00	0.99
	Cd9	**0.58***	0.00	-0.12	0.15
	Ctsd	**0.41***	0.00	-0.13	0.10
	Enpp2	**2.25***	0.00	**-2.03#**	0.00
	Eva1a	**0.37***	0.00	**-0.50#**	0.00
	Ezr	**0.44***	0.00	**-0.37#**	0.00
	Fxyd1	**0.59***	0.00	**-0.54#**	0.00
	Gadd45g	**0.34***	0.00	**-0.40#**	0.00
	Gfap	**0.73***	0.00	-0.14	0.22
	Ggta1	**0.68***	0.00	-0.13	0.40
	H2-D1	**0.56***	0.00	**-0.26#**	0.01
	H2-K1	**0.53***	0.00	**-0.34#**	0.00
	Hhatl	**0.33***	0.03	-0.23	0.14
	Id4	**0.42***	0.00	**-0.43#**	0.00
	Lamp2	**0.37***	0.00	**-0.19#**	0.03
	Mdk	**0.63***	0.00	**-0.69#**	0.00
	Mgst1	**0.49***	0.00	**-0.40#**	0.00
	Msx1	**1.35***	0.00	**-1.38#**	0.00
	Mt1	**0.57***	0.00	**-0.67#**	0.00
	Mt2	**0.81***	0.00	**-0.72#**	0.00
	Myoc	**0.34***	0.00	**-0.24#**	0.01
	Nfe2l2	**0.57***	0.00	**-0.34#**	0.00
	Nfkbia	**0.96***	0.00	**-0.85#**	0.00
	Pdgfd	**0.48***	0.00	**-0.31#**	0.03
	Sdc4	**0.66***	0.00	**-0.33#**	0.00
	Serpinf1	**0.51***	0.00	**-0.44#**	0.01
	Slc14a1	**0.36***	0.00	0.10	0.28
	Vim	**0.37***	0.00	**-0.22#**	0.03

The data are expressed as the log2 ratio. *$p<0.05$ versus the WT vehicle group; #$p<0.05$ versus the Tau22 vehicle group.

Supplemental Figures and Legends

Supplemental Figure 1

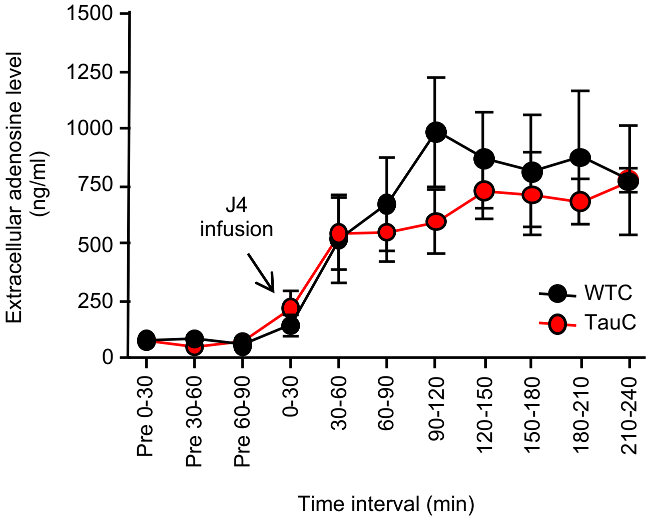

Supplemental Figure 1. J4 elevates extracellular adenosine levels in the hippocampus of Tau22 mice. Mice (Female, 11 months old, WTC, black, n=4; TauC, red, n=6) were subjected to *in vivo* microdialysis. The basal level of extracellular adenosine was measured for 90 mins before the infusion of J4 (1 mM, indicated by the arrow) into the hippocampus. Data are expressed as the mean ± S.E.M.

Supplemental Figure 2

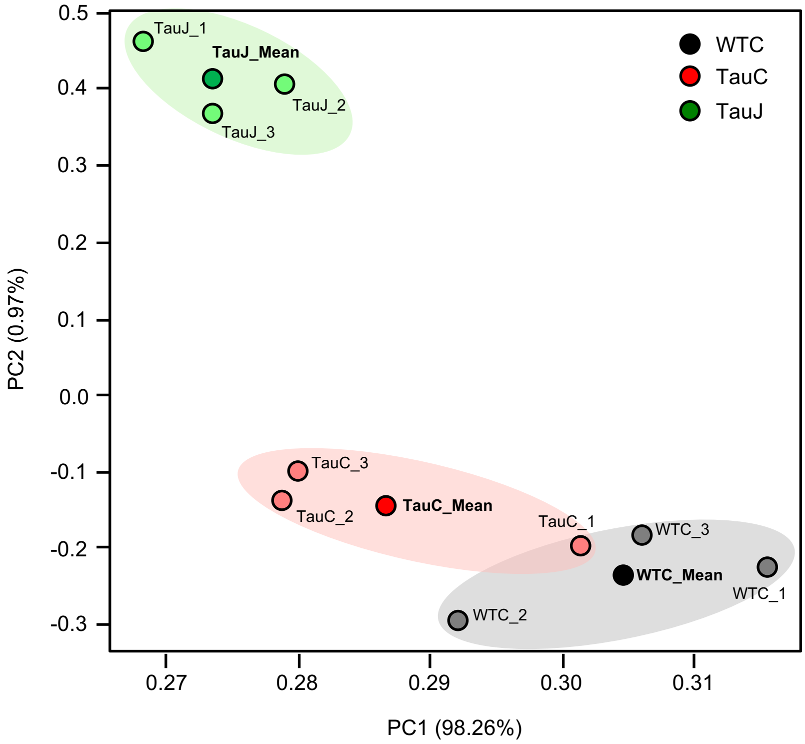

Supplemental Figure 2. Principal component analysis (PCA) of the RNAseq data in the current study. The PCA scatter plot of gene expression in the hippocampi of mice treated as indicated (WTC, black; TauC, red; and TauJ, green; n= 3 in each group) from the age of 3 to 10 months. The PCA plot shows the variance of the three biological replicates of each treatment group. Percentages on each axis represent the variance captured by principal component 1 (PC1) and principal component 2 (PC2).

Supplemental Figure 3

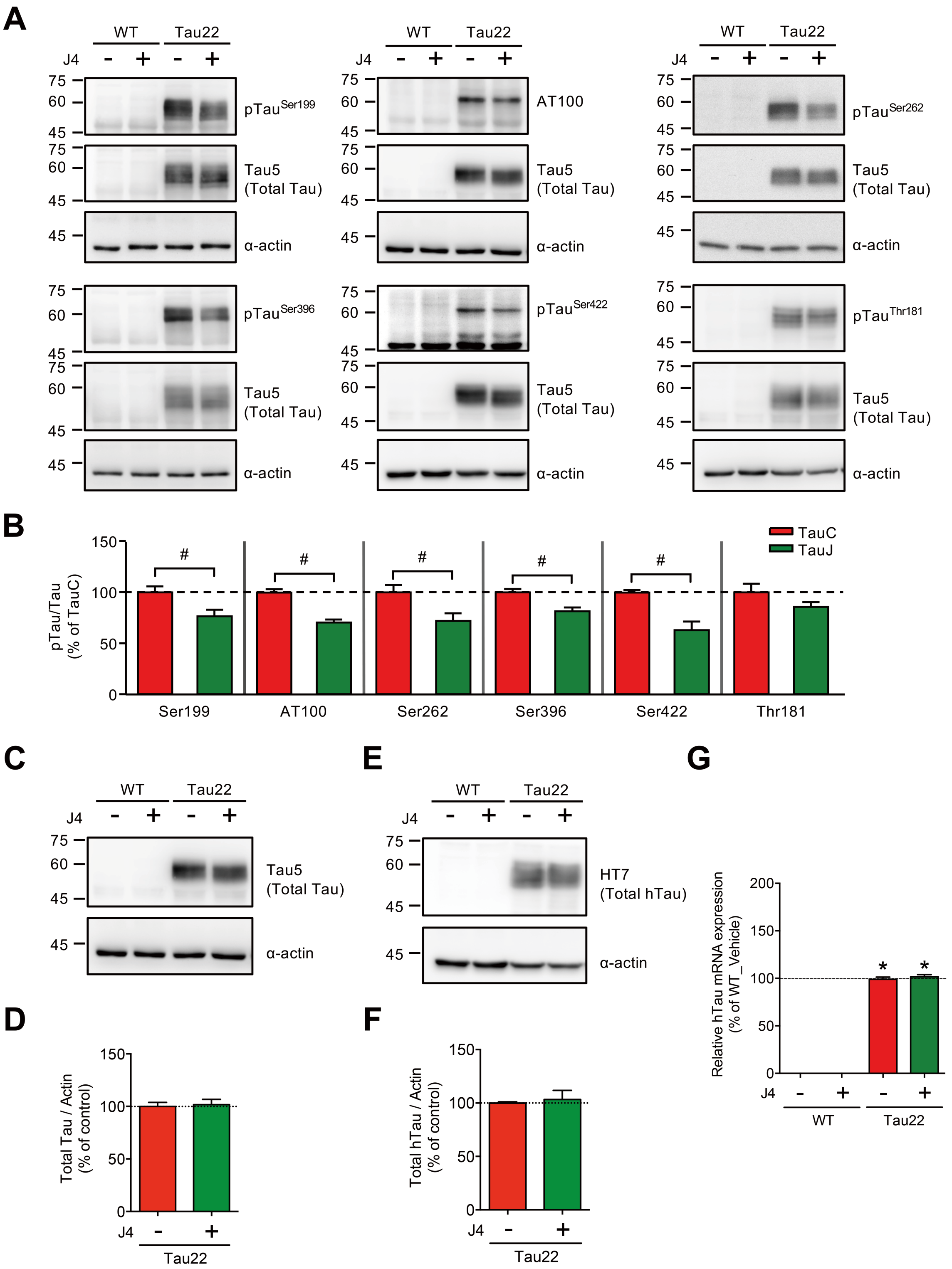

Supplemental Figure 3. Chronic J4 treatment decreases hyperphosphorylated Tau levels in the hippocampi of Tau22 mice and does not affect the expression of the human Tau protein and transgene. Mice were treated as indicated (control WT mice, WTC; J4-treated WT mice, WTJ; control Tau22 mice, TauC; and J4-treated Tau22 mice, TauJ n = 3 - 4 in each group) from the age of 3-11 months. (**A-F**) Total hippocampal lysates (20 μg per lane) were harvested and subjected to WB analysis. The protein expression levels of (**A**)

phospho-tau (pSer181, pSer199, pThr212/Ser214, pSer262, pSer396, and pSer422), (**C**) tau protein (Tau5), (**E**) human tau protein (HT7), and α-Actin (loading control) were detected using the indicated antibodies and the quantification results are shown in (**B**), (**D**), and (**F**), respectively. (**G**) The gene expression level of human Tau in mice from the different treatment groups (WTC, black; WTJ, blue; TauC, red; TauJ, green; n = 3-5) from the age of 3-11 months was examined by RT-qPCR. The data are expressed as the mean ± S.E.M. *$p < 0.05$ versus the WTC group; one-way ANOVA.

Supplemental Figure 4

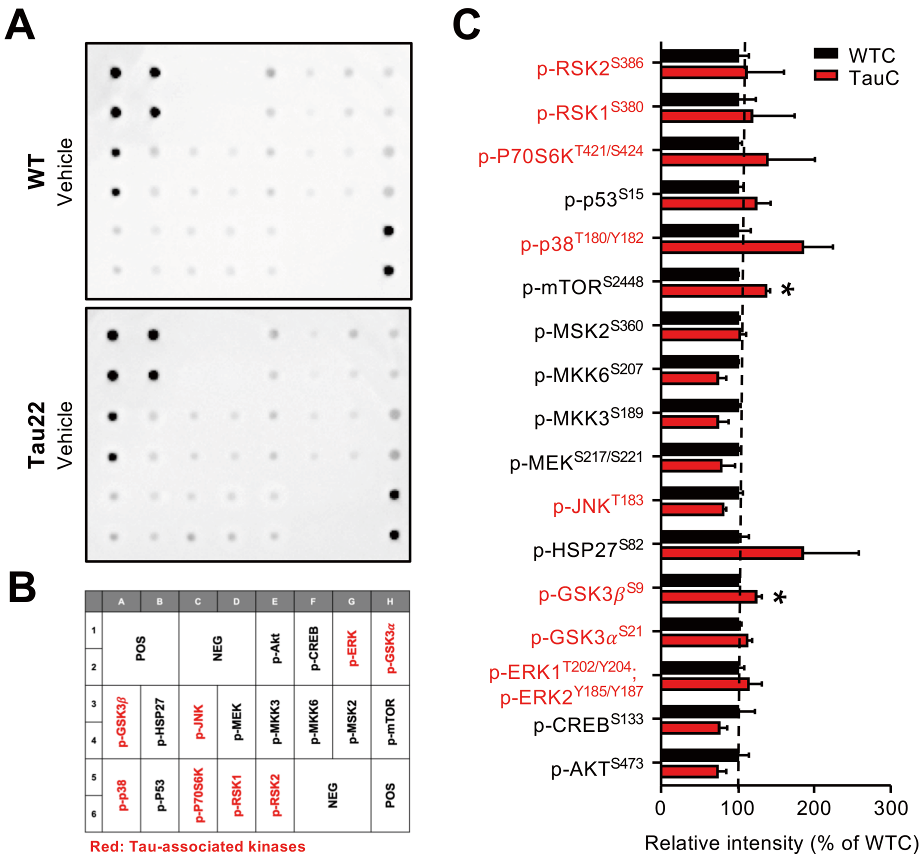

	A	B	C	D	E	F	G	H
1	POS		NEG		p-Akt	p-CREB	p-ERK	p-GSK3α
2								
3	p-GSK3β	p-HSP27	p-JNK	p-MEK	p-MKK3	p-MKK6	p-MSK2	p-mTOR
4								
5	p-p38	p-P53	p-P70S6K	p-RSK1	p-RSK2	NEG		POS
6								

Supplemental Figure 4. MAPK phosphorylation array analysis of Tau22 mice.

Mice were treated as indicated (control WT mice, WTC; and Control Tau22 mice, TauC; n = 6 in each group) from the age of 3-11 months. Pooled total hippocampal lysates (1 mg from 3 mice) were harvested and subjected to MAPK phosphorylation array. Four independent determinations were obtained from two independent sample preparations. Each preparation contains lysates from three mice. (**A**) The phosphorylation level of MAP kinases (including Tau-associated kinases) was assessed following the manufacturer's protocol. (**B**) The map of MAPK phosphorylation array. Words in red are Tau-associated kinases. Data from each array was normalized to the averaged positive control signals (POS) followed by being normalized to the WTC group. The relative intensity of indicated kinase is shown in (**C**). The data are expressed as the mean ± S.E.M. *p<0.05 versus the WTC group, two-tailed Student's t-test.

Supplemental Figure 5

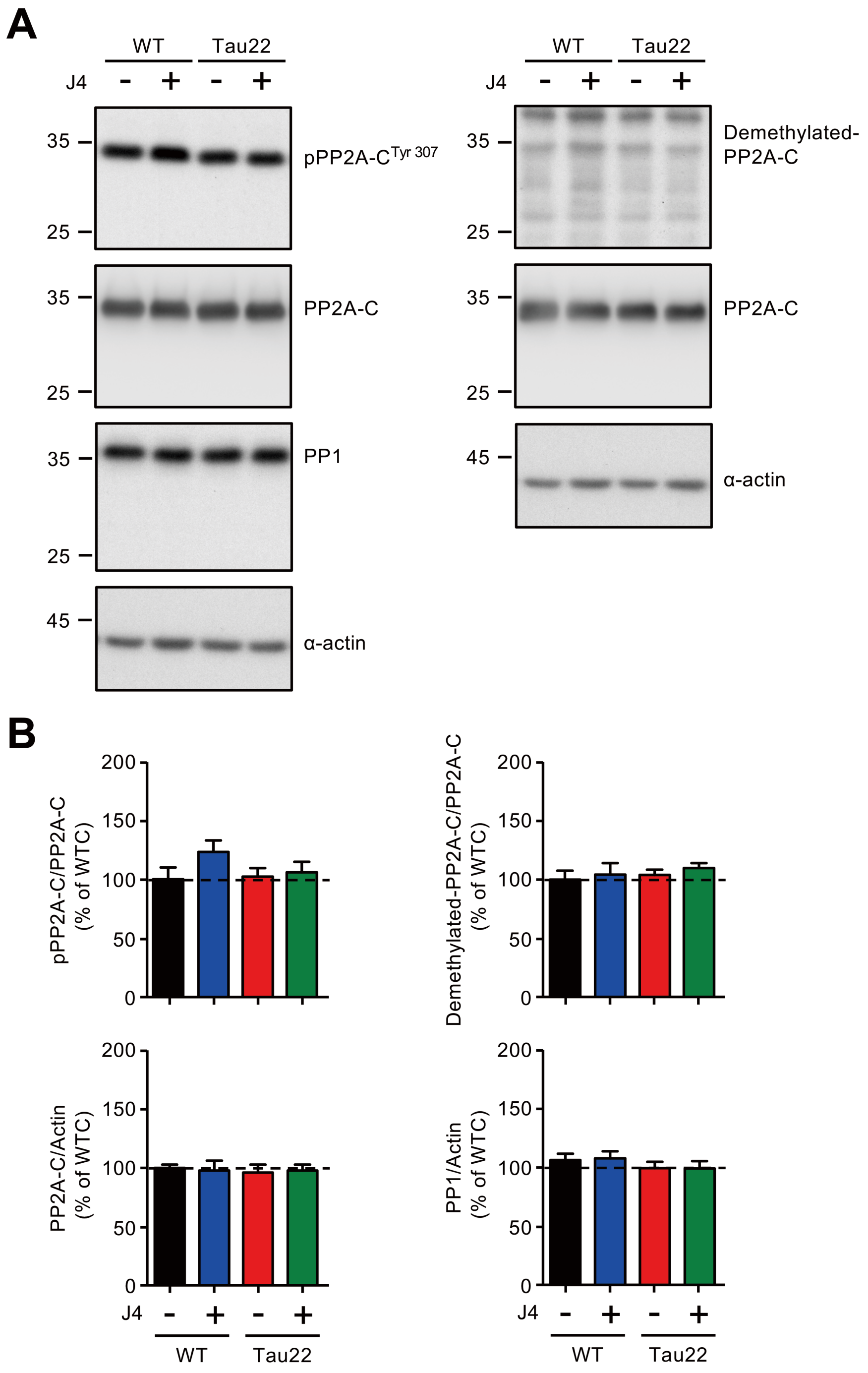

Supplemental Figure 5. Activities and expressions of PP1 and PP2A in Tau22 mice.

Mice were treated as indicated (control WT mice, WTC, black; J4-treated WT mice, WTJ, bue; control Tau22 mice, TauC, red; and J4-treated Tau22 mice, TauJ, green; n = 5-7 in each group) from the age of 3-11 months. (**A**) The total hippocampal lysates were harvested carefully, and subjected to Western blot analysis (30 µg per lane) for the amounts of phospho-PP2A-C (pTyr307), demethylated-PP2A-C, PP2A, and PP1 as indicated. The level of the indicated signal was normalized to that of α-Actin (a loading control) and shown in (**B**). The data are expressed as the mean ± S.E.M.

Supplemental Figure 6

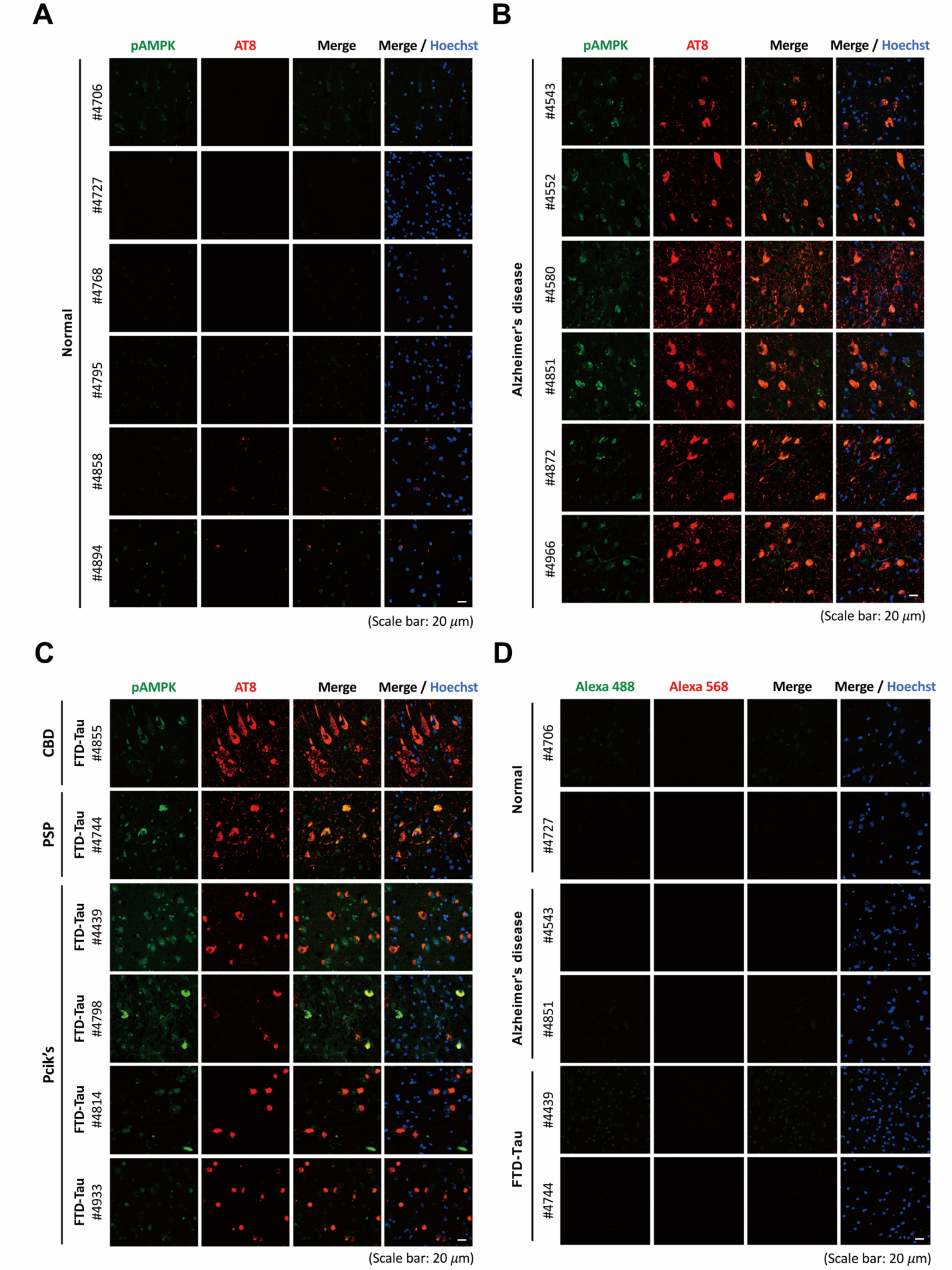

Supplemental Figure 6. AMPK activation can be observed in the posterior hippocampus of Alzheimer's

disease and FTD-Tau patients.

Posterior hippocampal sections (6 µm) from six normal subjects (**A**), six Alzheimer's disease patients (**B**), and six FTD-Tau (CBD, PSP, and Pick's) patients (**C**) were subjected to IHC staining. The level of phospho-AMPK and hyperphosphorylation tau was evaluated by staining with the indicated antibodies (pAMPK^{Thr172}, green; AT8 for pTau$^{Ser202/Thr205}$, red). The negative control is shown in (**D**). Scale bar, 20 µm.

Supplemental Figure 7

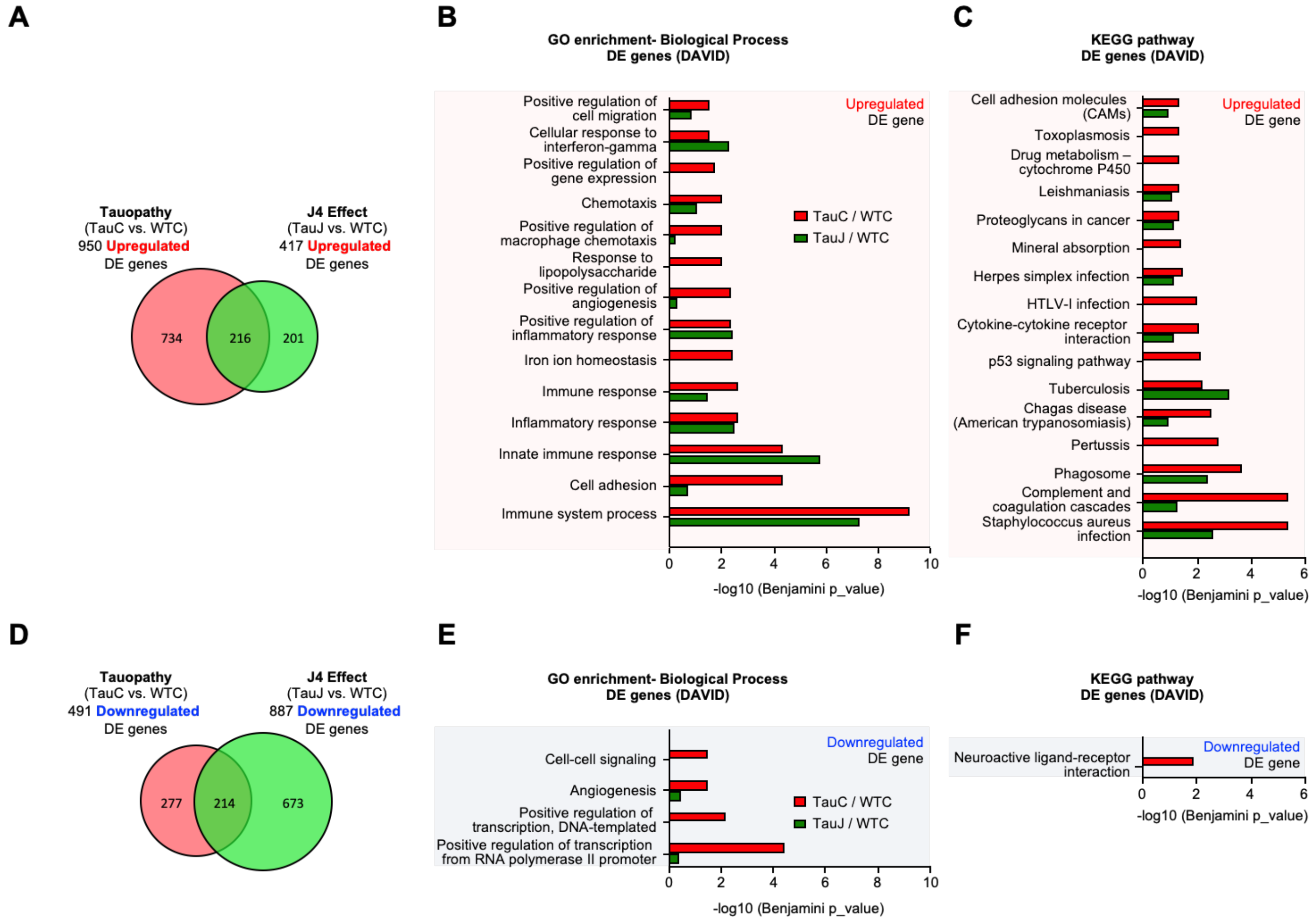

Supplementary Figure 7. Chronic J4 treatment lowers the significance of the tauopathy-associated GO term enrichment and KEGG pathway analysis in the hippocampi of Tau22 mice.

Mice were treated as indicated (WTC, TauC, and TauJ; n= 3 in each group) from the age of 3-10 months. The hippocampus was carefully removed for RNA-seq analysis. (A) The Venn diagram shows that 216 overlapping DE genes between the upregulated DE genes in the TauC group vs. WTC group (pink) and TauJ group vs. WTC group (green). (B) GO enrichment and (C) KEGG pathway analysis of the upregulated DE genes between the TauC and WTC groups (red bar) and TauJ and WTC groups (green bar). (D) Venn diagram showing the 214 overlapping DE genes between the downregulated DE genes in the TauC group vs. WTC group (pink) and TauJ group vs. WTC group (green). (E) GO enrichment and (F) KEGG pathway analysis of the downregulated DE genes between the TauC and WTC groups (red bar) and TauJ and WTC groups (green bar). The tauopathy-associated GO term and KEGG pathway were identified from the TauC group vs. the WTC group (FDR < 0.05, Benjamini $p < 0.01$).

Supplemental Figure 8

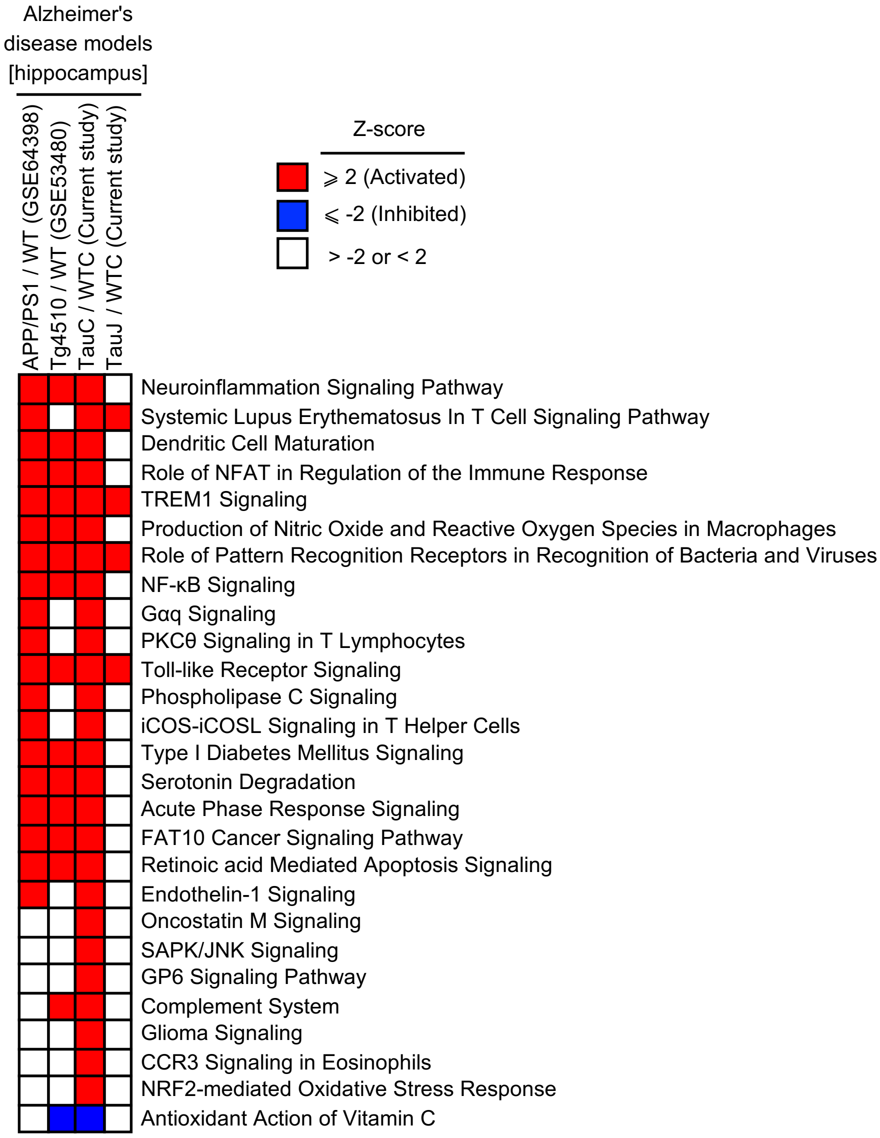

Supplementary Figure 8. J4 treatment reverses many dysregulated canonical pathways, also found in other mouse models of Alzheimer's disease, in Tau22 mice.

The heatmap shows the canonical pathways enriched in the hippocampus of Tau22 mice with or without J4 treatment and those in other Alzheimer's disease mouse models (Tg4510, GSE53480; APP/PS1, GSE64398). The 1441 DE genes of the TauC/WTC group and 1304 DE genes of the TauJ/WTC group were subjected to IPA-Analysis Match analysis. Z-score is used to predict pathway activation (Z-score ≥ 2.0, red) or inhibition (Z-score ≤− 2.0, blue).

Supplemental Figure 9

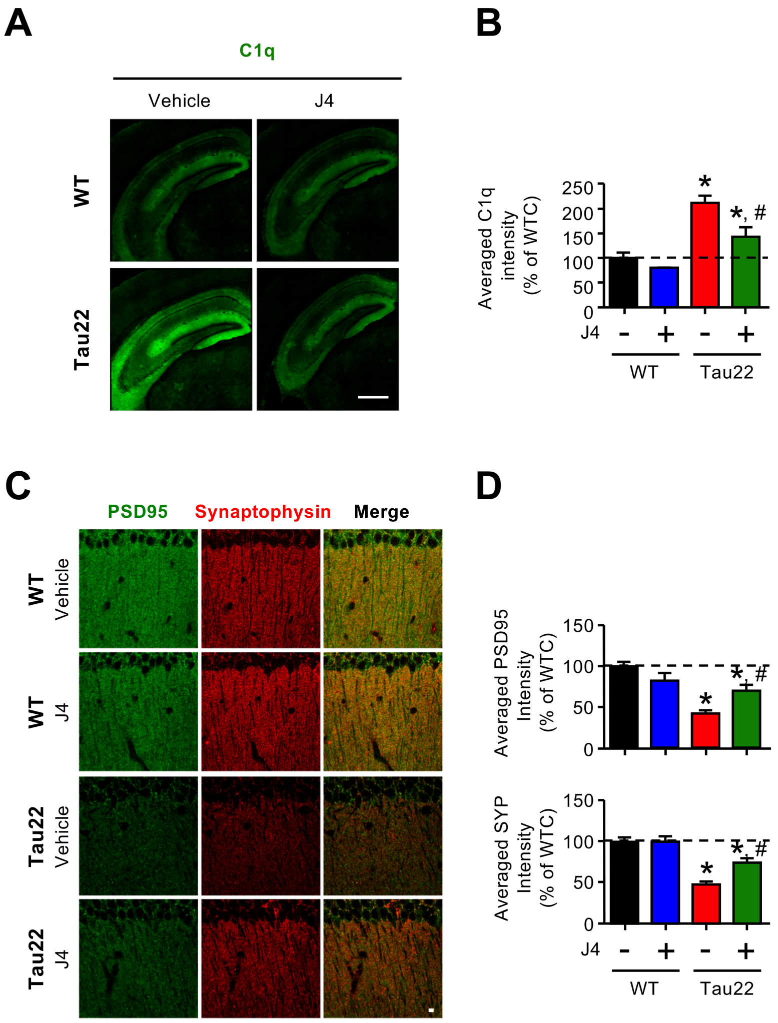

Supplemental Figure 9. Chronic J4 treatment reduces the C1q elevation and elevates the synaptic protein level in the hippocampi of Tau22 mice.

Mice were treated as indicated (WTC, black; WTJ, blue; TauC, red; TauJ, green; n = 5-7 in each group) from the age of 3-11 months, and their tissues were subjected to IHC staining. (**A**) The intensity of C1q (green) and Iba1 (red) expression was examined and the quantification results are shown in (**B**). Scale bar, 20 µm. (**C**) The intensity of synaptic marker expression in the hippocampi of treated mice (n=10-12 in each group) was evaluated by staining with the indicated antibodies (PSD95, green; SYP, red). The quantification results are shown in (**D**). Scale bar, 5 µm. The data are expressed as the mean ± S.E.M. *$p < 0.05$ versus the WTC group; $^{\#}p < 0.05$ versus the TauC group, one-way ANOVA.

Supplemental Figure 10

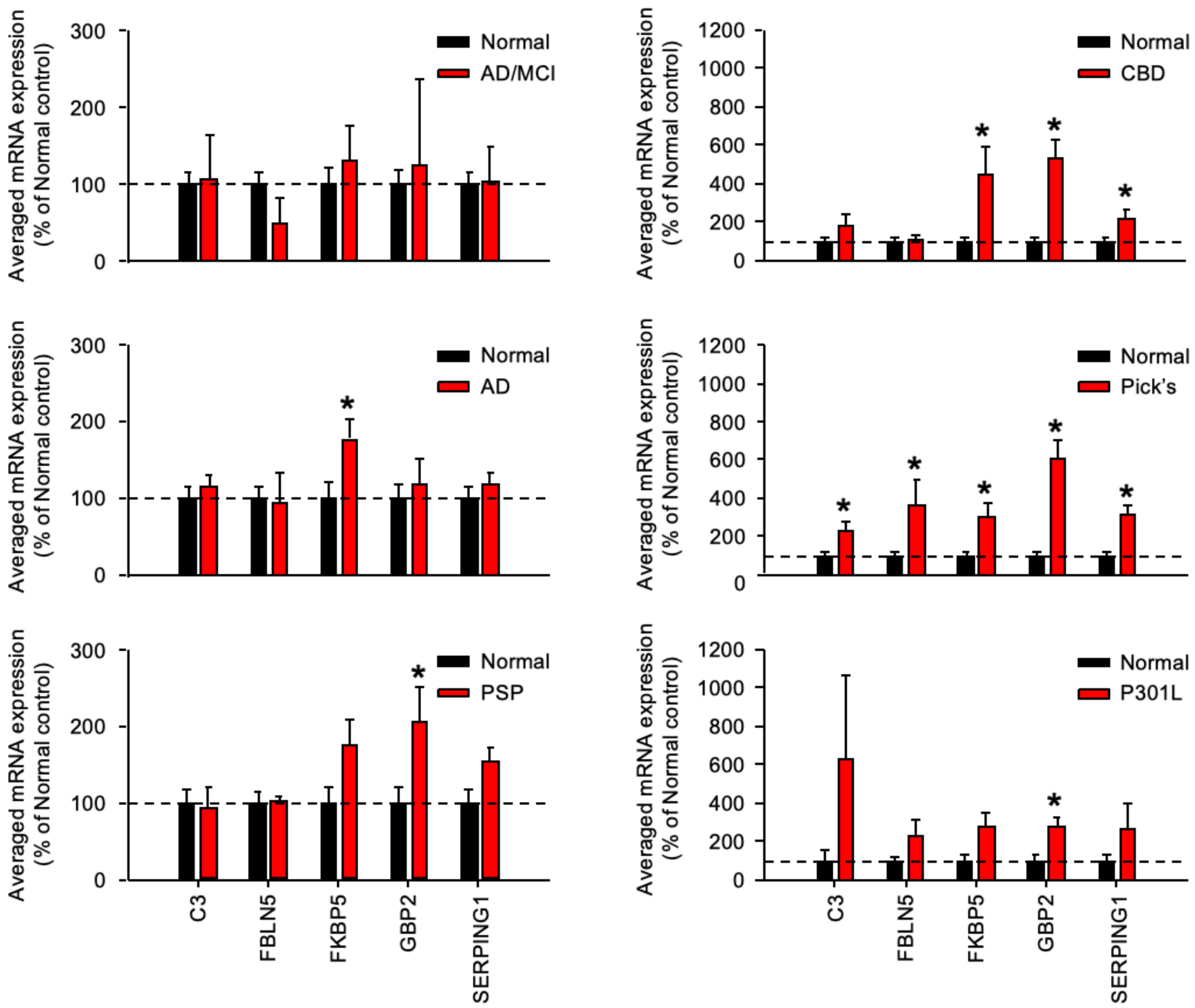

Supplemental Figure 10. The expression of the A1-specific genes in the frontal cortex of AD and FTD-Tau patients.

The expression levels of A1-specific genes in the frontal cortices of AD (AD/MCI, Braak 3-4, n=5; AD, Braak 5-6, n=14) and FTD-Tau patients (CBD, n=5; P301L, n=3; Pick, n=5; PSP, n=10) and normal control subjects (n=3-10) were analyzed by RT-qPCR. The relative expression level of genes is listed. ACTB was used as a reference gene for normalization. The data are expressed as the mean ± SEM. **p*<0.05 versus the normal control subjects; two-tailed Student's t-test. AD, Alzheimer's disease; FTD, Frontotemporal dementia; FTLD, Frontotemporal lobar degeneration; CBD, Corticobasal degeneration; PSP, Progressive supranuclear palsy.

Supplemental Figure 11

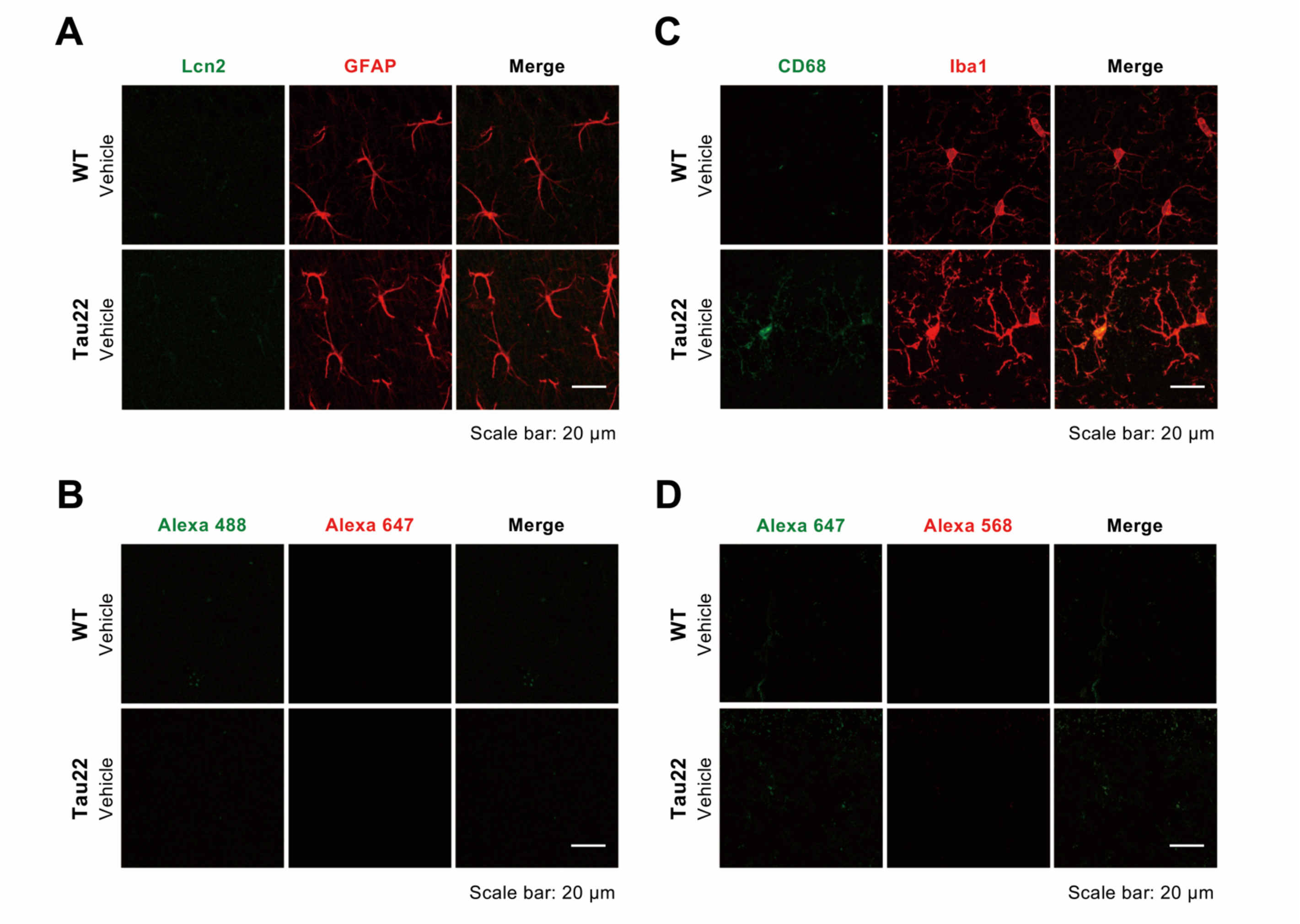

Supplemental Figure 11. Microgliosis, but not astrogliosis can be observed at 4 months of age in the hippocampus of Tau22 mice.

Hippocampal sections (20 μm) were prepared from Tau22 mice (n=3) and their littermate control (n=3) at the age of 4 months and subjected to IHC staining. The level and the activity of the (**A**) astrocyte and (**C**) microglia in the hippocampus were evaluated by staining with the indicated antibodies (GFAP for astrocyte, red; Lcn2 for reactive astrocyte, green; Iba1 for microglia, red; CD68 for reactive microglia, green), and the negative control are shown in (**B** and **D**). Scale bar, 20 μm.

Supplemental Figure 12

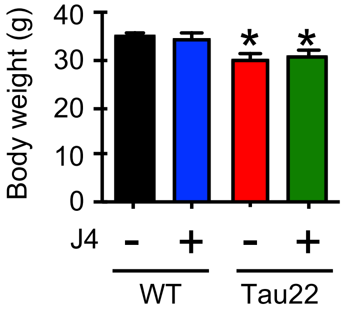

Supplemental Figure 12. J4 does not affect the body weight of Tau22 mice and their littermate controls (WT).

Mice were treated as indicated from the age of 3 to 11 months. The body weight of each animal was measured at the age of 11 months (WTC, black, n=25; WTJ, blue, n=22; TauC, red, n=27; and TauJ, green, n=23). Data are expressed as the mean ± S.E.M. *$p < 0.05$ versus the WTC group; one-way ANOVA.

Supplemental Figure 13

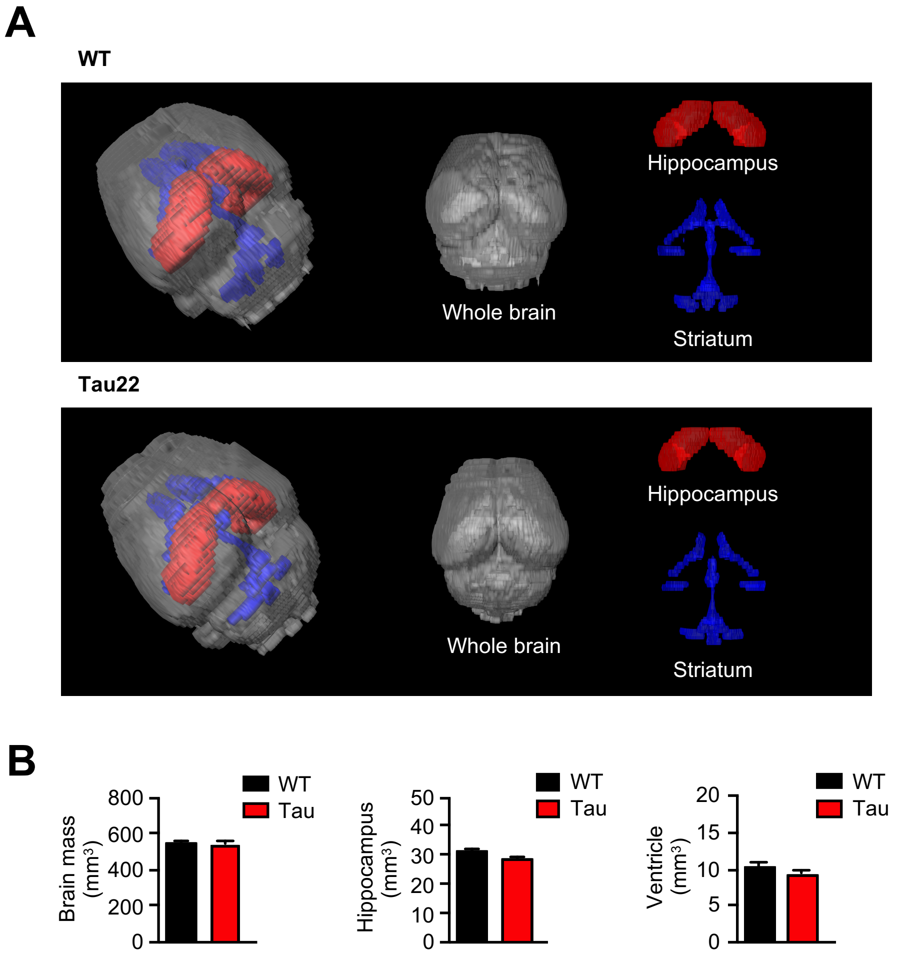

Supplemental Figure 13. No significant alteration in the volume of the whole brain, hippocampus, and ventricle is observed in Tau22 mice of 11 months.

Brain tissues were prepared from WT and Tau22 mice (n=3 in each group) at the age of 11 months, and subjected to micro-MRI imaging. (A) Representative 3D micro-MRI images of WT and Tau22 mice. From left to right, the reconstituted images represented the whole brain (grey), hippocampus (red), and ventricles (blue). (B) The volume (mm^3) of the whole brain mass, hippocampus, and ventricles of WT (black) and Tau22 mice (red) were measured. Data are expressed as the mean ± S.E.M.

Supplemental Figure 14

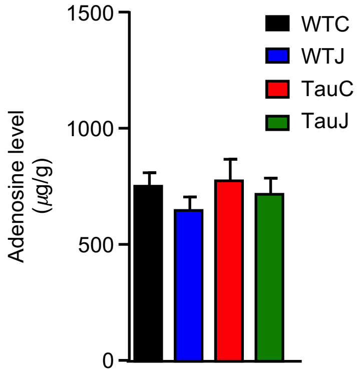

Supplemental Figure 14. The bulk adenosine levels in mouse hippocampi.
Total hippocampal lysates (2.5 μg) harvested from mice with the indicated treatment (WTC, black, n=4; WTJ, blue, n=5; TauC, red, n=5; and TauJ, green, n=5; 3-11 months) were subjected to HPLC analysis. The amount of adenosine was analyzed and quantified. Data are expressed as the mean ± S.E.M.

Supplemental Figure 15

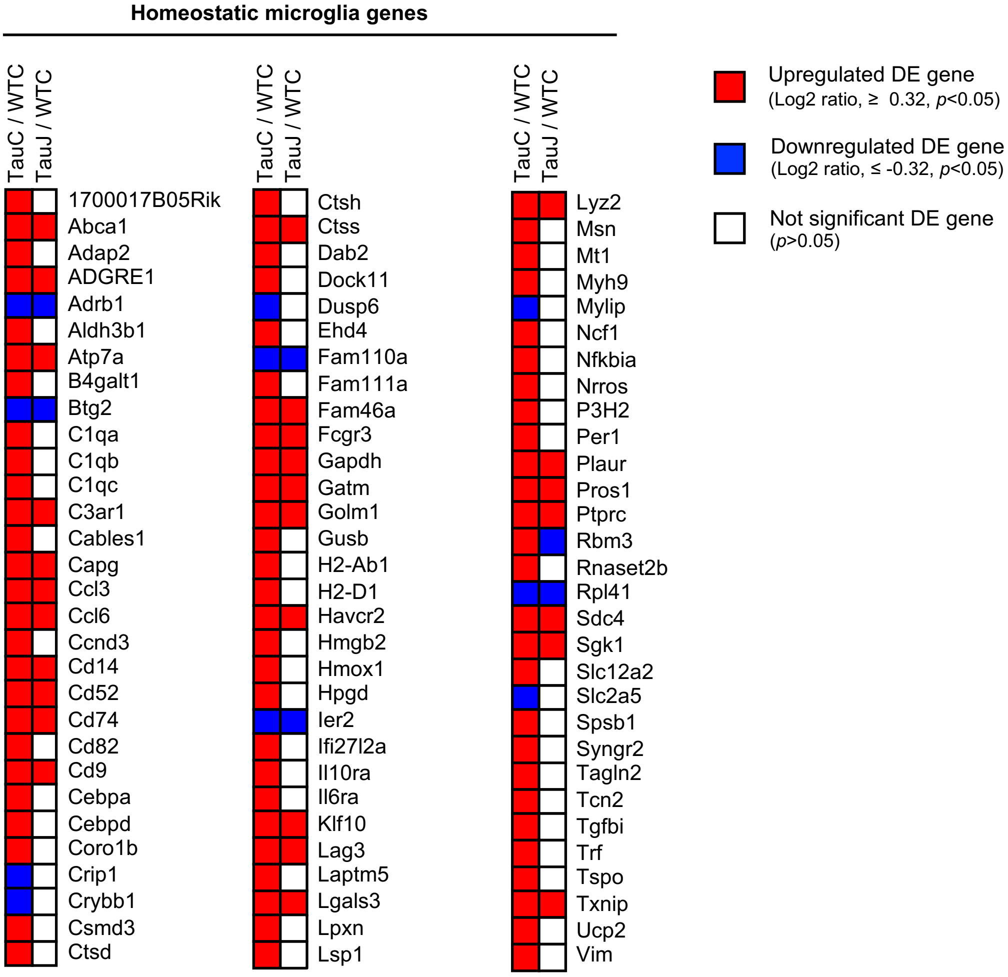

Supplemental Figure 15. Chronic J4 treatment reduces the dysregulated homeostatic microglia genes in the hippocampus of Tau22 mice.

Mice were treated as indicated (n= 3 in each group) from the age of 3 to 10 months. The hippocampus was carefully removed for RNA-seq analysis. Heatmap shows the relative expression level ($\log_2$ ratio) of the homeostatic microglia genes in the hippocampus of TauC/WTC and TauJ/WTC groups. The upregulated ($\log_2$ ratio ≥ 0.32; $p < 0.05$) and downregulated ($\log_2$ ratio ≤ -0.32; $p < 0.05$) DE genes are shown in red and blue, respectively. The genes with no statistical difference in their expressions between TauJ and WTC are shown in white.

Supplemental References

1 Bhatt DP, Chen X, Geiger JD, Rosenberger TA (2012) A sensitive HPLC-based method to quantify adenine nucleotides in primary astrocyte cell cultures. J Chromatogr B Analyt Technol Biomed Life Sci 889-890: 110-115 Doi 10.1016/j.jchromb.2012.02.005

2 Horman S, Browne G, Krause U, Patel J, Vertommen D, Bertrand L, Lavoinne A, Hue L, Proud C, Rider M (2002) Activation of AMP-activated protein kinase leads to the phosphorylation of elongation factor 2 and an inhibition of protein synthesis. Curr Biol 12: 1419-1423 Doi 10.1016/s0960-9822(02)01077-1

3 Lee CC, Chang CP, Lin CJ, Lai HL, Kao YH, Cheng SJ, Chen HM, Liao YP, Faivre E, Buee Let al (2018) Adenosine Augmentation Evoked by an ENT1 Inhibitor Improves Memory Impairment and Neuronal Plasticity in the APP/PS1 Mouse Model of Alzheimer's Disease. Mol Neurobiol 55: 8936-8952 Doi 10.1007/s12035-018-1030-z

4 Li S, Xiong GJ, Huang N, Sheng ZH (2020) The cross-talk of energy sensing and mitochondrial anchoring sustains synaptic efficacy by maintaining presynaptic metabolism. Nat Metab 2: 1077-1095 Doi 10.1038/s42255-020-00289-0

5 Woods A, Dickerson K, Heath R, Hong SP, Momcilovic M, Johnstone SR, Carlson M, Carling D (2005) Ca2+/calmodulin-dependent protein kinase kinase-beta acts upstream of AMP-activated protein kinase in mammalian cells. Cell Metab 2: 21-33 Doi 10.1016/j.cmet.2005.06.005
